# Will we ever be able to accurately predict solubility?

**DOI:** 10.1038/s41597-024-03105-6

**Published:** 2024-03-18

**Authors:** P. Llompart, C. Minoletti, S. Baybekov, D. Horvath, G. Marcou, A. Varnek

**Affiliations:** 1 Laboratory of Chemoinformatics, UMR7140, University of Strasbourg, Strasbourg, France; 2 IDD/CADD, Sanofi, Vitry-Sur-Seine France

**Keywords:** Cheminformatics, Physical chemistry

## Abstract

Accurate prediction of thermodynamic solubility by machine learning remains a challenge. Recent models often display good performances, but their reliability may be deceiving when used prospectively. This study investigates the origins of these discrepancies, following three directions: a historical perspective, an analysis of the aqueous solubility dataverse and data quality. We investigated over 20 years of published solubility datasets and models, highlighting overlooked datasets and the overlaps between popular sets. We benchmarked recently published models on a novel curated solubility dataset and report poor performances. We also propose a workflow to cure aqueous solubility data aiming at producing useful models for bench chemist. Our results demonstrate that some state-of-the-art models are not ready for public usage because they lack a well-defined applicability domain and overlook historical data sources. We report the impact of factors influencing the utility of the models: interlaboratory standard deviation, ionic state of the solute and data sources. The herein obtained models, and quality-assessed datasets are publicly available.

## Introduction

Aqueous solubility is a strategic parameter in synthetic, medicinal and environmental chemistry. It is one of the main parameters affecting bioavailability. Thus, a better understanding of this property is expected to improve success in drug design^1^, as a key player in pharmacokinetics and ADME-Tox (Absorption, Distribution, Metabolism, Excretion, and Toxicity) profiling^2^. Solubility governs the fraction of the active substance available for absorption in the gastro-intestinal tract. Besides, a poor solubility of a compound or of a metabolite can be a threat for the patient: the substance may accumulate and crystalize, as exemplified by kidney stone diseases. Galenic formulation can improve the therapeutic potential of a compound^3^, but a soluble drug candidate is always a safer option for clinical trials.

However, measuring aqueous solubility is not always feasible at the early discovery stage because of the low throughput and large sample requirements^4,5^. For this reason, *in silico* predictive approaches have become highly valuable to prioritize drug candidates and reduce the number of experimental tests. Latest progress in this field is mainly due to (i) the organization of aqueous solubility prediction challenges, shedding a new light on existing tools; (ii) the public release of large aqueous solubility datasets; (iii) the advent of new machine learning methods promising unprecedented predictive performances. The current *status quo* in solubility prediction, which this study aims to analyze, is therefore very intricate.

In the first part of this study, we first remind the theoretical background of aqueous dissolution process, underlining the ambiguities and complexity of this measure. Next, we review the large number of datasets already published. Third, we critically discuss published models. This enables us, in a second part, to propose new guidelines to process thermodynamic aqueous solubility data. We applied them to existing datasets and proceed to a modeling exercise resulting in new QSAR models. All curated datasets and obtained models are publicly available at 10.57745/CZVZIA^6^.

### Background of aqueous solubility

Several types of solubility measurements are reported in the literature, depending on the method and conditions of measurement. The *thermodynamic solubility* is described as the maximum concentration of a compound in solution, at equilibrium with its most stable crystalline form. This solubility is usually measured during lead optimization phases and is used as source of *in silico* regression models^7^. However, the above definition is not unambiguous, as the solute may, beyond physically dissolving, also *chemically* interact with water – with significant impact on the equilibrium. Therefore, no less than three distinct “thermodynamic” solubility measures are being used: water, apparent and intrinsic. The *water solubility* is measured with pure water as the added solvent. At equilibrium, the solution is a mixture of the potentially many proteolytic microspecies of the solute, and the sum of their concentration counts as “water solubility”. Acid-base interactions induce self-buffering effects, stabilizing the solution at a specific pH value, which must be reported as well. By contrast, the *apparent solubility* is defined in a fixed-pH buffer solution; it is also called *buffer solubility* and reflects the relative population of dissolved microspecies at the buffer pH. Finally, the *intrinsic solubility* (S_0_) is the maximum concentration of the neutral compound: the pH of the solution is adjusted so the non-ionized compound becomes the predominant microspecies. Under certain assumptions and approximations, the Henderson-Hasselbalch (HH, Eq. (3) equation estimate the aqueous solubility (S), from the intrinsic solubility (S_0_), the acidity or basicity constant (pK_a_ or pK_b_), and the pH^8^. Additionally, the *kinetic solubility* is often preferred during the early phase of drug discovery at the screening platforms level. It is frequently described as the lowest concentration at which the species starts to precipitate when diluting a 10 mM DMSO stock solution in buffer, usually Phosphate-Buffered Saline (PBS) 7.4. The kinetic solubility is usually perceived as a crude estimate of the thermodynamic solubility. Although these values are related, they quantify distinct phenomena: in kinetic measurements, there is no control or knowledge of the precipitating crystalline or amorphous form^9^, and artefacts due to supersaturation cannot be excluded. Additionally, there may exist large variations in the experimental setup between providers of kinetic solubility values; as a result, many of them cannot be used together^9^.

Accurately predicting thermodynamic solubility remains a challenge as numerous physicochemical and thermodynamic factors are involved. Some of them are, the solid-solvated phase transition, solid state (amorph or crystal), temperature, polymorphism, intermolecular interactions between solute-solvent and the co-occurring ionic forms of electrolytes^10^. Even though numerous drugs are electrolytes, they are still hard to predict at specific pH as their aqueous solubility is the result of co-occurring microspecies^11,12^. Over the past decades, several approaches have been developed to early identify poorly soluble compounds.

#### Experimental techniques

To ensure high quality data, experiments should use pure substance, temperature control and sufficient time for the solute to reach equilibrium. The current OECD 105 Guideline for the testing of chemicals^13^ recommends two approaches for measuring thermodynamic water solubility: (i) the shake-flask method for chemicals with a solubility above 10 mg/L (ii) the column elution or slow-stir method for chemicals with solubilities below 10 mg/L.

The shake-flask method consists of mixing a solute in water until the thermodynamic equilibrium between the solid and solvated phase is reached. Then, the two phases are separated by either centrifugation or filtration. The column elution method consists of pumping water through a column coated with the chemical. The water flows at a constant rate through the column and is recirculated until equilibrium. For each method, the concentration of compound in the filtrate is measured to obtain the thermodynamic solubility. When working with surfactants, the slow-stir method should be used. Surfactants are amphiphilic organic compounds highly miscible in water. However, agitation and high concentration can induce micelle formation, distorting the measurements. This concentration point is called the Critical Micelle Concentration (CMC). The slow-stir avoids emulsion and helps solubilize low-density compounds using a controlled magnetic stirring.

An advanced technique called CheqSol was suggested by Llinas *et al*.^14^. Developed by Stuart *et al*.^15^ to establish thermodynamic equilibrium conditions during measurement, the technique can measure the intrinsic and kinetic solubility of ionizable compounds. It is an automated titration method where the pH is adjusted until the solute precipitate or until the precipitate dissolves itself. The concentration of uncharged species is deduced from the point of equilibrium and the pK_a_; this process is called Chasing Solubility. The method works down to 1 mg/L and is restrained to mono- and di-protic compounds with known pK_a_ / pK_b_.

#### Limit of detection and quantification

The LoQ is the lowest possible concentration of an analyte that can be quantified by the method with precision and confidence. The LoD is the lowest concentration at which the method can detect. Thus, LoQ defines the limits associated to a 95% probability of obtaining correct value. Their determination is important as they define the sensitivity of the analytical method used. Thus, using measurements lower than the LoD or LoQ present higher probability of error. Compounds labeled “below LoD/LoQ” may not be used in regression models as their effective solubility is not precisely known but are safe to be labeled as “insoluble” in categorical models.

#### Dataset description

**Fig. 1:**
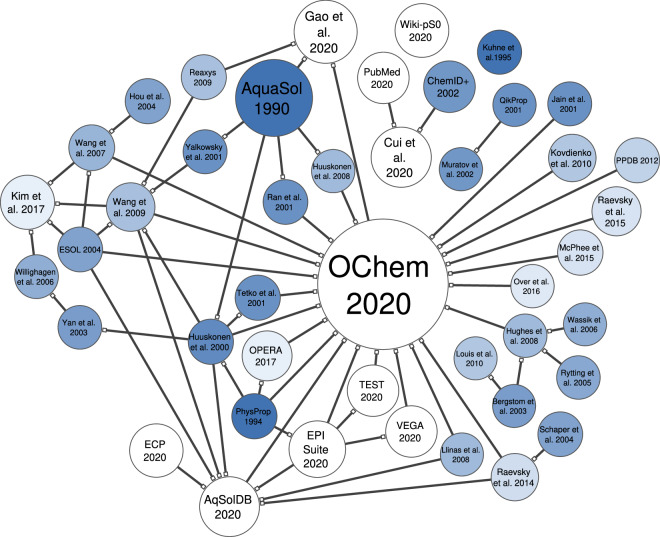
Network of the reported thermodynamic aqueous solubility datasets. Supersets composed by merging of previously available datasets are connected to the latter by directed edges, on which a hollow square connector designs the superset. For example, Raevsky *et al*.^132^ includes Schaper *et al*.^133^, and is included in both OChem2020, and AqSolDB2020. The node size defines the number of entries of the datasets. The node color defines the age of the dataset, from dark blue (old) to white (recent). ECP stands for eChemPortal, and ChemID + states ChemIDPlus.

Thermodynamic solubility data sets gather these measurements and property prediction. Over the years, the ensemble of data has continued to grow to now reach more than 20 libraries available online, some of them containing more than 50,000 entries, Fig. 1. Depending on their source, experimental conditions such as the temperature (T°C), pH, cosolvents and others may be reported. These metadata should also be taken in account when refining data for modeling.

These libraries largely overlap, drawing a very complex network of relationships. Numerous modelers have used the dataset of Huuskonen *et al*.^16^ from 2000, which gathers entries from AquaSol^17^ and PhysProp^18^. AquaSol was published in 1990 by Yalkowsky *et al*., reporting almost 20,000 records for 6,000 compounds. By that time, it was the most extensive compilation of thermodynamic solubility measurements for unionized compounds. Before that, PhysProp, published in 1994 by Syracuse, was the first large set containing values for 1,297 organic compounds. The ESOL^19^ library, was disclosed in 2004 by Delaney; it contains 2,874 measurements for both ionized and unionized compounds.

As of now, these sets are still widely used and found in other libraries such as EPI Suite^20^, Wang *et al*.^10^ from 2007, Wang *et al*.^21^ from 2009 and Kim *et al*.^22^ from 2020. Reporting recent measurements, their size ranges from 1,676 entries for Wang *et al*. from 2007, to 8,031 entries for EPI Suite. Fusion of datasets into ever growing supersets raises the problem of proper management of “duplicate” entries. If both merged sets independently include the same experimental value taken from a same source, trivial duplication of the entry should be imperatively avoided, when there is a risk of having one item in the training set and its identical in the validation set. This concern EPI Suite 2009, ESOL 2004, OPERA 2018, Tetko *et al*.^23^ and Huuskonen *et al*.^16^. Moreover, it appears that the actual types of solubility reported by the sets differ. Some, such as Wiki-pS0 of 2020 and Llinas *et al*. of 2008 only contain *intrinsic solubility* entries. Llinas *et al*.^14^ of 2008 reports 105 measurements available online. They were obtained using the CheqSol technique and used during the Solubility Challenge 2 (SC2). Wiki-pS0^24^ is a private database of drug-like compounds owned by in-ADME research. As of 2009, Wiki-pS0 contained 6,355 entries for 3,014 unique compounds. Entries were obtained from CheqSol measurements, or through the conversion of aqueous to intrinsic solubility using pDISOL-X.

However, other datasets like AqSolDB^25^ and OChem^26^ are undefined mixtures of *intrinsic*, *apparent* and *water* solubility data. They now represent the largest thermodynamic solubility repositories freely available. OChem is an online platform reporting properties measurements linked to scientific articles and offering a modelling interface. As of September 2022, OChem “Water Solubility” (property = 46, in the OChem database structure) dataset contains 51,602 entries for almost 15,000 compounds and different solubility types, labeled as intrinsic solubilities. It also contains a dataset of “Water Solubility at pH” (property = 363, in the OChem database structure). The database aggregates entries from almost 150 sources, federating most of today’s measurements. However, it remains rarely used by the community, with only three applications for aqueous solubility data in 2021–2023, by Panapitiya *et al*.^27^, Wiercioch *et al*.^28^, and Lowe *et al*.^29^. In comparison, AqSolDB which was published in 2020 has already been used in 2021 by Francoeur *et al*.^30^ and Sluga *et al*.^31^, in 2022 by Meng *et al*.^32^ and Lee *et al*.^33^, and in 2023 by Lowe *et al*.^29^. AqSolDB is one of the largest publicly accessible set with 9,982 entries. It compiles nine open-source data sets. AqSolDB is known to have measurements of quality obtained from liquid, solid, or crystallized substances. Due to their diversity in solubility types, conditions and measurement techniques, these datasets require thorough curation to be used for modeling.

Yet, some sets remain poorly shared or used by the community. In particular, this concerns PubMed, QikProp^34^, ChemIDplus^35^, Khune *et al*.^36^ of 1995, eChemPortal^37^ and Wiki-pS0. eChemPortal provide free public access to information on the properties of chemicals. Most of them are part of ECHA REACH^38^, within which details about experimental conditions, protocol and substance composition can be found. ChemIDplus is a database containing information from the Toxicology Data Network. It contains chemical records of drugs, pesticides, pollutants, and toxins. Although relatively vintage, these datasets are overlooked resources that contain a wealth of experimental data.

#### Solubility prediction

Predictive approaches are either based on theorical equations or Machine Learning (ML) methods, including Neural Networks (NN). The few approaches based on first principles are mainly applied to estimate the solvation energy changes associated with a solute transitioning from its solid state to its solvated state.

From a thermodynamic point of view, solubilization can be managed in one or two steps starting from a solid material. It can either be by sublimation from solid to gas or by fusion from solid to liquid, followed with an energy transfer to water. Hence, in 1965 Irmann^39^ coupled the entropy of fusion (Δ*S*_m_) to the melting point (MP) through a group contribution approach to predict water solubility. Then, in 1968, Hansch *et al*.^40^ found that the water solubility of organic liquid compounds was linearly dependent to the octanol/water partition coefficient (Log *P*_o/w_). Yalkowsky *et al*.^41^ combined these results in 1980 to develop the General Solubility Equation (GSE) and estimate the base-10 logarithm of water solubility Log_10_*S*_*w*_ using the MP and Log *P*_o/w_ - see Eq. (1).

1 $$Lo{g}_{10}({S}_{w})=0.5-0.01\cdot (MP-25)-Log{P}_{o/w}$$

The equation is restrained to solid nonelectrolytes, but it usually performs well (RMSE: 0.7–0.8 log) when employed with experimental values^42^. Here, an electrolyte is a chemical substance that produces mobile charges. As most drugs are electrolytes, only few are covered by the GSE. Also, High Throughput Screening (HTS) does not usually include the measurement of MP and Log *P*_o/w_, which are thus replaced by predicted values. Their use can introduce major discrepancies in the estimation of thermodynamic solubility, not to mention that the prediction of MP represents itself a challenge. Thus, the GSE is not practically useful for large-scale predictions.

### 20 years of solubility modelling

Most of today’s models are Quantitative Structure Property Relationship (QSPR). These methods seek to find a mathematical function expressed as Y = f(X) where X defines a set of N molecular descriptors [D_1_, D_2_, …, D_N_] to correlate to the response value Y. Of course, the inner representation of a chemical graph by a GNN (Graph Neural Network) is no different. In our case, this Y value is the base-10 logarithm of the molar measurement of thermodynamic solubility, expressed as $Lo{g}_{10}\left(S\right)$.

Machine learning methods are mainly used to develop regression models leveraged on the compound’s topological, electronic, structural 2D/3D features, and molecular fragment counts. Models are then optimized using many ML methods to best fit the descriptors set. Recently, feature-based NN, graph-based NN (GNN) and structural attention methods have been used to develop powerful solubility predictive models. Tables 1 to 3 report a representative but not exhaustive list of aqueous solubility models developed over the last 20 years. It aims to highlight significant trends and achievements in this area. While the table includes models using diverse methods, caution is advised regarding overly optimistic performances. Depending on the data and approach employed, three periods can be distinguished. Prior to 2008, models were trained on vast datasets such as AquaSol, PhysProp and their aggregation, Huuskonen *et al*.^16^ (Table 1). Few methods (ANN, SVM, MLR and theorical equations) were applied as the most decisive parameter of one’s ML model performance was the size and diversity of its training set. From them, two lessons can be shared:

- The relationship between solubility and the classical descriptors used here tends to be largely non-linear. Therefore, in this context, ANNs clearly outperformed linear regression.
- The prediction performances are limited by the quality of the experimental data. It is usually measured using the Inter-laboratory Standard Deviation (*SDi*) - Eq. (2). It is considered as a lower limit for theoretical prediction accuracy, and it was pointed out that the *SDi* can reach up to 1.0 log unit.

2 $$SDi=\sqrt{\frac{{\sum }_{i=1}^{n}{\left({x}_{i}-\bar{x}\right)}^{2}}{n-1}}$$

**Table 1:** Reported performances of the thermodynamic solubility models published from 1997 to 2007.

Year	Reference	Descriptors	Size	Dataset	Method	RMSE	R2
**1997**	Huuskonen *et al*.^87^	Electrotopological / Topological	83	Litterature	ANN	—	0.84
**2000**	Huuskonen *et al*.^16^	Structural	694	Khune *et al*.	MLR	—	0.67
							0.87
					ANN		0.85
							0.84
**2001**	Tetko *et al*.^23^	Molconn-Z	1,291	Huuskonen *et al*.	MLR	0.81	0.85
					ANN	0.66	0.9
	Ran *et al*.^42^	Melting Point / cLogP	380	AquaSol	GSE	0.76	—
	Bruneau^88^	2D/3D/Charge/ Katrizky	2,233	Huuskonen *et al*.	ANN	0.82	—
	Liu *et al*.^89^	2D Topological	1,312	Huuskonen *et al*.	ANN	0.71	—
**2002**	Klamt *et al*.^90^	QM	257	QikProp dataset	MLR	0.61	—
	Engkvist *et al*.^91^	1D/2D Descriptors	1,290	Huuskonen *et al*.	ANN	—	0.95
	Chen *et al*.^92^	Dipole, PSA, Vol, MW, Rot. & H-acc/don and D	321	Litterature	MLR	0.86	0.71
**2003**	Wegner & Zell^93^	2D Topological	1,290	Huuskonen *et al*.	ANN	0.54	—
	Cheng & Merz^94^	Cerius	2,440	AquaSol, PhysProp, Merck Index, PDR, CMC	MLR-GA	1.01	—
	Yan & Gasteiger^95^	PETRA	1,293	Huuskonen *et al*.	MLR	—	0.89
					ANN		0.94
	Lind & Maltseva^96^	Electrostatic, QM & topological	1,296	Huuskonen *et al*.	SVM	0.68	0.89
**2004**	Yan *et al*.^97^	PETRA	2,084	Huuskonen *et al*.	ANN	—	0.94
	Hou *et al*.^98^	2D Topological	1,299	Huuskonen *et al*.	MLR	—	0.9
	Fröhlich *et al*.^99^	MOE & JOElib	1,297	Huuskonen *et al*.	SVM	—	0.9
	Votano *et al*.^100^	Fragments & Counts	4,115	Aquasol, Physprop, PDR, Taskinen, Tetko, Lobell	MLR & PLS	—	0.84
					ANN		0.84
			1,840		ANN		0.86
	John S. Delaney^19^	cLogP, MW & Count	2,874	Abraham, Pesticide Manual, Syngenta	ESOL	—	0.55
**2005**	Matthew Clark^101^	2D descriptors	3,724	PhysProp	PLS	—	0.84
	Catana *et al*.^102^	MOE, E-state & ISIS key	1,107	Pfizer proprietary & Public	PLS	0.48	0.94
					Non-Linear PLS		
					NN		
**2006**	Hansen *et al*.^43^	MOE 2D/3D	4,569	PhysProp	ANN	0.97	0.94
	Wassvik *et al*.^103^	Tm, LogP, Sm, Hm & Molconn-Z	428	Astrazeneca	GSE	0.92	0.73
					Mod. GSE	0.73	0.78
**2007**	Wang *et al*.^10^	3D Topological, cLogP, MW & Count	1,878	Delaney *et al*., Huuskonen *et al*., Hou *et al*.	MLR	0.74	0.9
	Johnson *et al*.^45^	VOLSURF	362	Literature	MLR & HH	0.61	0.88
	Schwaighofer *et al*.^104^	Dragon	1,290	Huuskonen *et al*.	GP	0.55	0.93
			4,597	Huuskonen *et al*. & Others		0.55	0.91

ANN: Artificial Neural Network

ASE: Abraham Solvation Equation

CNN: Convolutional Neural Network

CPANN: Count-Propagation Artificial Neural Network

DNN: Deep Neural Network

D-GIN: Directed GIN

D-MPNN: Directed-MPNN

GIN: Graph Isomorphism Network

GP: Gaussian Process

GNN: Graph Neural Network

GSE: General Solubility Equation

HH: Henderson-Hasselbalch equation

KNN: Kernel Neural Network

LS-SVM: Least-Square Support Vector Machine

MAT: Molecule Attention Transformer

MK: Multi Kernel

MLR: Multi Linear Regression

MLR-GA: Multi Linear Regression Genetic Algorithm

MPNN: Message Passing Neural Network

NFP: Neural FingerPrint

NL-PLS: Non-Linear Partial Least Square

PLS: Partial Least Square

RF: Random Forest

RM: Replacement Method

SMILES: Simplified Molecule Input Line Entry System

SNN: Shallow Neural Network

SR: Stepwise regression

SVM: Support Vector Machine

SVR: Support Vector Regression

TE: Theorical Equation

UG-RNN: Undirected Graph Recurrent Neural Network

CR: Contracted Ring

LMO: Leave-Many-Out

LOO: Leave-One-Out

**Table 2:** Reported performances of the thermodynamic solubility models published from 2008 to 2014.

Year	Reference	Descriptors	Size	Dataset	Method	RMSE	R2
**2008**	Cheung *et al*.^105^	MOE	110	Litterature	MLR	—	0.9
					ANN		0.85
	Duchowicz *et al*.^106^	Dragon	166	Merck Index	RM	—	0.85
	Huuskonen *et al*.^48^	DayLight	191	AquaSol, Merck Index, ChemFinder & PhysProp	MLR	—	0.8
	Hughes *et al*.^107^	cLopP & Tm	237	Bergström *et al*., Rytting *et al*. & Wassvik *et al*.	MLR	1.03	0.63
					SVM		
	Zhou *et al*.^49^	ECFP	1,299	Huuskonen *et al*.	PLS	0.71	0.85
	Husskonen *et al*.^48^	cLogP & Counts	365	AquaSol	MLR	—	0.87
	Du-Cuny *et al*.^108^	LogP, Fragments & Index	2,473	Roche proprietary	PLS	0.42	0.84
	Obrezanova *et al*.^109^	ATC, logP, Volume & MW	592	Syracuse	GP	0.71	0.88
**2009**	Wang *et al*.^21^	ATC,ClogP, MW	4,874	Delaney *et al*. & Huuskonen *et al*.	MLR	0.98	0.83
	Hewitt *et al*.^53^	LogP, Tb & Dragon	104	SC1	MLR	0.95	0.74
					ANN	1.51	0.79
	Duchowicz & Castro^110^	Dragon	145	Merck Index	MLR	0.9	0.76
**2010**	Ghafourian & Bozorgi^111^	ACD-Labs & TSAR 3D	141	Rytting *et al*.	SR	0.71	—
	Muratov *et al*.^112^	2D Simplex	290	Klampt *et al*.	PLS	—	0.81
	Cao *et al*.^65^	Dragon	225	Llinas *et al*. & Merck Index	SVR	—	0.74
	Jain & Yalkowsky^113^	Activity coefficients, Melting Entropy & MP	883	AquaSol & EPA	TE	—	0.73
	Eric *et al*.^114^.	CODESSA	319	Rytting *et al*.	MLR	0.96	0.66
	Louis *at al*^115^	Marvin & Karselson	74	Bergstrom *et al*. & others	MLR	0.8	0.55
					ANN	0.74	0.59
					SVM	0.83	0.53
	Fatemi *et al*.^116^	LFER from ADME Boxes	145	Duchowicz *et al*.	MLR	0.92	0.71
					LS-SVM	0.73	0.85
					ANN	0.75	0.72
**2012**	Chevillard *et al*.^64^	MOE, ADMET predictor & ISIDA	4,897	PhysProp, Huuskonen *et al*. & SC1	RF	0.51	0.62
						0.72	0.56
						0.89	0.23
	Slavica *et al*.^50^	CODESSA	374	Eric Slavica *et al*.	CPANN	0.68	—
**2013**	Lusci *et al*.^47^	2D Graph	1,144	Delaney *et al*.	UG-RNN	0.58	0.92
					UG-RNN-CR	0.79	0.86
					UG-RNN + logP	0.61	0.91
					UG-RNN-CR + log P	0.63	0.91
					2D kernel	0.61	0.91
	Salahinejad *et al*.^117^	VOLSURF, CPSA, Energy lattice and Sublimation enthalpie	4,376	PhysProp	MLR	—	0.9
**2014**	McDonagh *et al*.^118^	CDK	100	CSD	PLS	1.08	—
					RF	0.93	
					SVR	1.17

**Table 3:** Reported performances of the thermodynamic solubility models published from 2015 to 2023.

Year	Reference	Descriptors	Size	Dataset	Method	RMSE	R2
**2017**	Kim *et al*.^119^	RDKIT	1,676	Willighagen *et al*., Wang *et al*. & Delaney *et al*.	Multi-kernel	0.61	0.91
	Coley *et al*.^120^	Undirected 2D graph	1,144	Delaney *et al*.	SVM	1.12	—
					CNN	0.56	
**2018**	Goh *et al*.^54^	SMILES	1,128	ESOL	DNN	0.63	—
	Cho *et al*.^121^	2D Graph & 3D bond features	270	ESOL	3DGCN (DNN)	0.66	—
					Weave (DNN)	0.78	
					NFP (DNN)	0.79	
**2019**	Cho *et al*.^122^	Atoms features	270	ESOL	GCN	0.63	—
**2020**	Deng & Jia^123^	2D Graph	1,128	Delaney *et al*.	DNN	1	0.78
					SNN	1	0.73
					RNN	0.97	0.72
					CNN	1.05	0.73
					ESOL	0.94	0.78
	Boobier *et al*.^22^	CDK	100	DLS-100	MLP	0.99	0.71
		—		—	HUMAN	0.94	0.72
	Gao *et al*.^124^	3D Graph	2,874	Delaney *et al*.	MGCN	0.13	0.99
					SchNet	0.1	0.99
			694	Huuskonen *et al*.	MGCN	0.05	0.99
					SchNet	0.05	0.99
	Cui *et al*.^55^	Fingerprints	9,943	ChemIDplus, PubMed & Litterature	ResNet CNN	0.68	0.41
	Alex Avdeef^24^	AbSolv and RDKIT	3,014	Wiki-pS0	GSE	1.17	0.6
					ASE	1	0.71
					RF	0.6	0.89
	Sluga *et al*.^48^	Dragon & MD topological	9,982	AqSolDB	ANN	0.59	0.93
					MLR	1.22	0.58
	Falcon-Cano *et al*.^125^	RDKit & Alvascience	9,982	AqSolDB	RF	0.73	0.72
**2021 to 2023**	Wiercioch *et al*.^28^	2D Graph	1,311	OChem	GNN	0.59	—
	Shen *et al*.^126^	2D Graph	1,128	ESOL	CNN (MolMapNet)	0.58	—
	Tosca *et al*.^127^	ChemGPS	270	Litterature	ANN	0.97	0.42
					GSE	1.12	0.22
					ANN	1.18	0.7
					GSE	1.2	0.69
	Wieder *et al*.^128^	2D Graph	5,216	Delaney *et al*.	D-GIN	0.8	—
					D-MPNN	0.86	
					GIN	1.09	
					RF	0.76	
					SVM	0.73	
					KNN	1.06	
	Chen & Tseng^129^	SMILES	1,128	Delaney	CNN	0.56	0.96
	Panapitiya *et al*.^130^	Mordred, ED Features, Rdkit & NWChem	17,149	Gao et al. & Cui *et al*.	MDM	1.05	0.77
					GNN	1.07	0.76
					SMILES	1.14	0.73
					SCHNET	1.23	0.69
	Francoeur *et al*.^30^	2D Graph	9,893	AqSolDB	MAT	1.71	0.68
	Meng *et al*.^32^	2D Graph	1,128 to 30,099	AquaSol, PhysProp, ESOL, OChem & AqSolDB	ChemProp	0.52	—
					AttentiveFP	0.59	
	Panapitiya *et al*.^27^	3D Graph, 3D/2D Descriptors & Fragments	11,868	Gao *et al*.	MDM	1.05	0.77
					GNN	1.07	0.76
					SMILES	1.14	0.73
					SCHNET	1.23	0.69
	Hou *et al*.^131^	SMILES	9,943	Cui *et al*.	BCSA	0.8	0.88
					GCN		
					AttentiveFP		
					MPNN		
	Lee *et al*.^33^	2D-Graph & Molecular FP	12,849	AqSolDB, ONSC, AAT & BNNLap	LightGBM	0.96	0.8
	Lowe *et al*.^29^	PaDEL	8,037	ADDoPT, AqSolDB, Bradley, eChemPortalAPI, LookChem, OChem, OPERA, PubChem, QSARDB	RF	0.97	0.82

The *SDi* depends of the average value $\bar{x}$ of the *n* replicated measures, *x*_*i*_.

Few attempts were also made to predict^43^ the *intrinsic* solubility using the HH equation. An ANN was trained on PhysProp to obtain the predicted aqueous solubility. Acidity and basicity constants (pK_a_ and pK_b_) required by HH were estimated by pKaPlugIn from ChemAxon^44^. The HH equation depends on the ionization state of the compounds and can thus be used by Hansen’s combined model to compute the *intrinsic* solubility ($Log\left({S}_{0}\right)$) as a function of pH – see Eq. (3).

3 $$Log\left({S}_{w}\right)=Log\left({S}_{0}\right)+\left(1+1{0}^{\left(pH-p{K}_{a}\right)}+1{0}^{\left(p{K}_{b}-pH\right)}\right)$$

In 2007, Johnson *et al*.^45^ renewed this approach by postulating an *ansatz* describing the *intrinsic* solubility as a function of the pK_a_, pK_b,_ pH and, crystal packing ${\chi }_{pack}$ and degree of ionization *F*_*I*_ – see Eq. (4). The influence of the crystal lattice on the solubility were simulated by a molecular dynamics simulation^45^.

4 $$Log\left({S}_{pH}\right)=Log\left({S}_{0}\right)+\min \left[Log\left(1{0}^{\mathop{\sum }\limits_{i}^{{N}_{acids}}\left(pH-pK{a}_{i}\right)+\mathop{\sum }\limits_{j}^{{N}_{bases}}\left(pK{b}_{j}-pH\right)+1}\right),4.25\right]-{\chi }_{pack}\cdot {e}^{-{F}_{I}}$$

It should also be noted that:

- Solubility is an equilibrium between solute-solvent interactions and crystal formation. Yalkowsky *et al*.^41^ proposed to use the melting point in the GSE as an early attempt to integrate crystal lattice effects. As MP depends on the polymorph, this approach is sensitive to polymorphism of solutes. So, the GSE requires either an experimental knowledge of the MP of the solutes or a precise knowledge of the polymorph. In both cases, it may be easier to measure the solubility directly.
- Additionally, the solubility of a compound is highly dependent on its acid-base properties, particularly when the solution pH is within 2 log units of the compound’s pK_a_. Any errors in estimating pKa can lead to large deviations in solubility values. Thus, it may be safer to rely on experimental determination for these properties rather than trying to estimate them in QSPR models.

The abundance of modeling approaches motivated Llinas *et al*.^14^ to organize in 2008 the *Solubility Challenge* (SC1). Its goal was to correctly predict the intrinsic solubility from 32 compounds using a given training set of 100 compounds. The challenge data covered a wide and high range in measurements, from 0.5 to 3.0 log unit. To predict it, participants used the full range of existing methods. Models’ performances highlighted difficulties in the prediction of highly and poorly soluble compounds. Overall, only about one-third of the compounds were correctly predicted by the best performing models, with the lower RMSE around 0.6 log^46^. SC1 sparked debates on how to enhance the predictive methods as well as the quality of the measurements. It also triggered the development of numerous models by the community, for which estimating the quality of the data took precedence over enhancing accuracy.

These methods employed novel neural network architectures (Table 2). For instance, Lusci *et al*.^47^ introduced in 2013 a method based on Undirected Graphs (UG). Their approach was applied with a 10-fold internal Cross-Validation (CV) to ESOL, Llinas *et al*. 2008, and Huuskonen *et al*.^16^ and reached a low RMSE of 0.58 log. Number of other approaches were introduced during this period: MLR by Huuskonen *et al*.^48^ in 2008, PLS by Zhou *et al*.^49^ in 2008, MLR by Wang *et al*.^21^ in 2009 and CPANN by Eric *et al*.^50^ in 2012.

This raise of powerful machine-learning methods available motivated Llinas and Avdeef^51^ to organize a second *Solubility Challenge* (SC2) in 2019. This time, they invited participants to apply their own models to 2 datasets. Set 1 consisted of 100 druglike compounds with an average *SDi* of 0.17 log. Set 2 contained 32 molecules with an average *SDi* of 0.62 log. Participants were asked to use their own training set. No significant improvements were found compared to the SC1^52^. Every method worked equally well and achieved a minimal RMSE of 0.70 log^14,51,53^.

The current period is marked by a trend of deep learning architecture and molecular embedding inputs emerged (Table 3). In 2018, Goh *et al*.^54^ introduced SMILE2vec, the first interpretable DNN to use SMILES for chemical property prediction. The developed NN was inspired by Word2Vec, a DL technique commonly used in NLP research. By comparing the performance of different Bayesian optimization techniques for hyperparameter tuning on the ESOL dataset, they were able to identify the most effective architecture, CNN-GRU. Applied to ESOL validation set, their model achieved a RMSE of 0.63 log and demonstrated interpretability by highlighting chemical functions, using a residual NN as a mask to identify important characters from the input. Their model accuracy outperformed feature-based methods.

A similar approach was conducted by Cui *et al*.^55^ in 2020 by adapting the well-known ResNet to accept PubChem fingerprints as input. They constructed N-layers (N = 14, 20, or 26) CNN models based on the architecture of ResNet. Models were evaluated with a 10-fold CV on 9,943 compounds from ChemIDplus and PubMed. They achieved a RMSE of 0.68 log, highlighting the advantage of going deeper. However, this is in contradiction with Francoeur *et al*.^30^ results from 2021, concluding that smaller networks performed better.

In their study, Francoeur *et al*. optimized a Molecular Attention Transformer (MAT) to predict aqueous solubility from SMILES representation, called SolTranNet. Their method is based on the MAT architecture developed by Maziarka *et al*.^56^ MAT functions by applying self-attention to a molecular graph where each node is defined as a feature vector. Vectors are then combined with the adjacency matrix before being fed to the NN layers. The MAT hyperparameters were optimized by minimizing the RMSE of an AqSolDB subset. To validate their model, SolTranNet was applied to three different test sets: the SC2 test set, Cui *et al*. 2020 dataset, and Boobier *et al*.^22^ 2017 dataset, resulting in RMSE values of 1.295, 0.813 and 0.845 log, respectively. SolTranNet has comparable performance to current ML models. However, Francoeur *et al*.^30^ points out that the small size of the community test sets limits the conclusions to be drawn from their reported performances. Even when trained over large sets, models may not be generalizable to other datasets, especially those from specific domains, such as compounds of pharmaceutical interest, as also mentioned in Lovrić *et al*.^57^.

We hypothesized that the performances published might be optimistic, because of: (i) inaccurate delimitation or failure from the applicability domain, if defined, and (ii) lack of independent external validation sets. Yet, caution is warranted when comparing model efficacy across studies, given the significant variability in test sets and methodologies. As of now, numerous models are still published without validation on completely independent sets. Different validation strategies, such as internal and external, can be distinguished, varying in levels of rigor. Internal validation makes use of the same data from which the model was fitted. External validation requires an independent dataset to correctly assess the model’s reproducibility and generalizability, and thus application to other chemical spaces (CS). However, it’s a common misconception that splitting a dataset into a training and a validation set (random split or k-fold CV) is sufficient, especially with GNN where data leakage can happen. Data leakage occurs when information from the test set is used in the training process, which can lead to biased performance assessment of the model. In CV, the test sets are independent to some extent^58^ but the training set largely overlap. In the case of GNN, this can happen if the GNN has seen test set chemical structures during the pre-training process. This problem has been discussed in various studies, offering alternative validation techniques as potential solutions^59^. Despite these criticisms, the efficacy of cross-validation remains undiscussed, as empirically demonstrated in works by Breiman & Spector^60^ and further supported theoretically by Vapnik^61^. The importance of the test set size, coverage and quality is supported by Francoeur *et al*.^30^. Ideally, this set should be meaningful and be excluded from the model training to ensure realistic performances. For instance, Cui *et al*. in 2020 validated their DNN models on two small test sets of 62, and 5 compounds, obtaining RMSE of 0.681 and 0.689 *LogS* unit, respectively. These test sets are arguably small, but the former was aggregated from recent literature while the second was composed of new in-house data. In this publication, models’ performances were also compared to human expert performances. This contrasted with previously reported results in Boobier *et al*. in 2017. In this study, models were trained and tested on 100 compounds from the DLS-100 dataset which regroup S_0_ entries, mostly from Llinas *et al*. 2008 and Rytting *et al*.^62^. Data were used following a train/test split of 75/25 compounds. As a result, humans performed equally as ML models with a RMSE of 1.087 for the former against 1.140 log for the later.

## Results

### Data

For this study, we used two public thermodynamic solubility datasets: AqSolDBc (our clean version of AqSolDB) and OChem. Our intent was to externally validate models trained on AqSolDBc by testing them over public data. Datasets are resumed in the Table 4.

**Table 4:** List of datasets and their sizes used for building and validating models. AqSolDBc is a clean version of AqSolDB and OChem is a public dataset.

Datasets	Size
AqSolDBc	8,047
OChem	7,463
*Shared with AqSolDBc*	5,212
*Specific to OChem*	2,251

### Chemical space maps

**Fig. 2:**
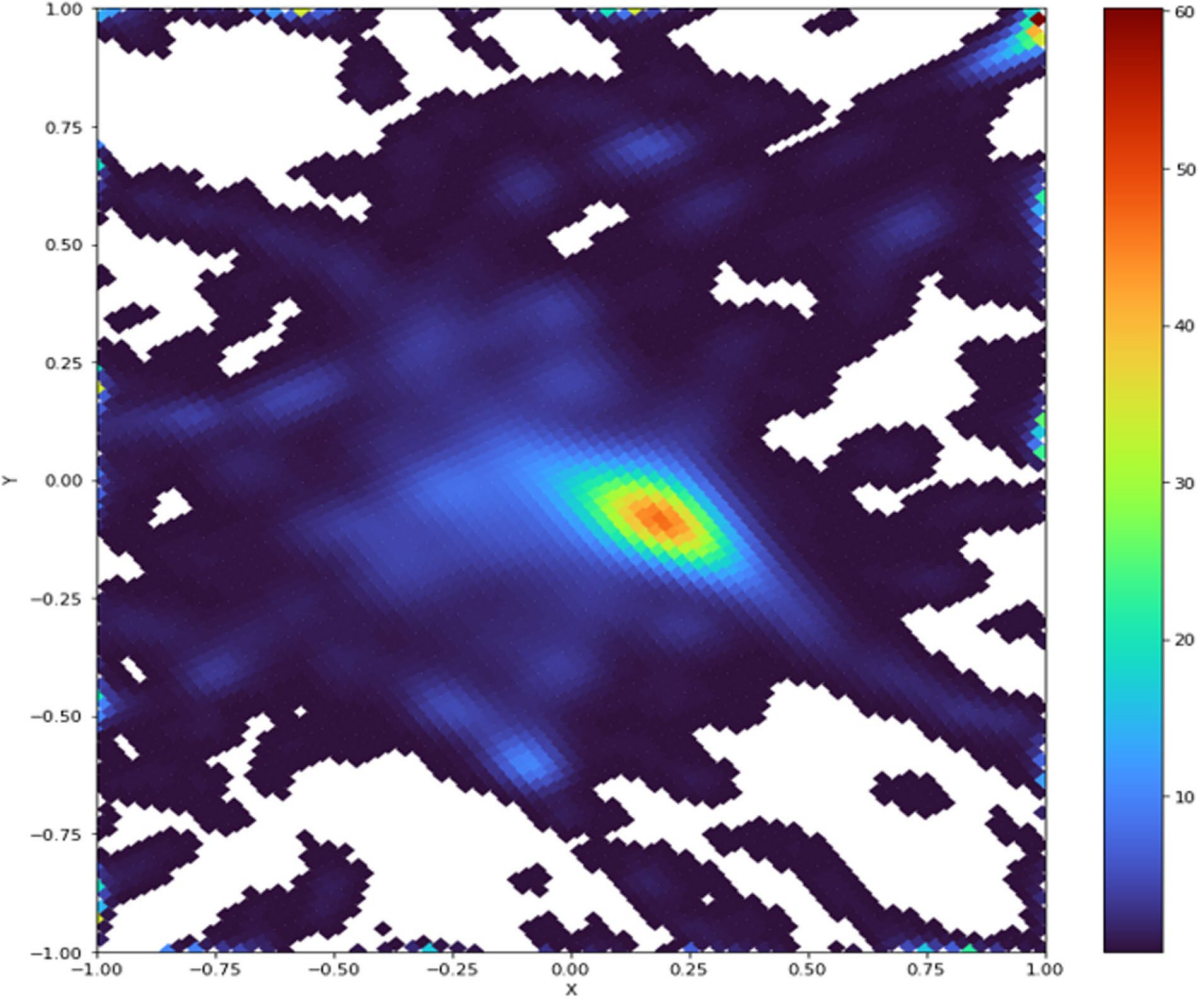
GTM density landscape of the chemical space jointly covered by AqSolDBc and OChem. White spaces are unpopulated areas. Colors represent the number of molecules per nodes, from blue (low) to red (high).

**Fig. 3:**
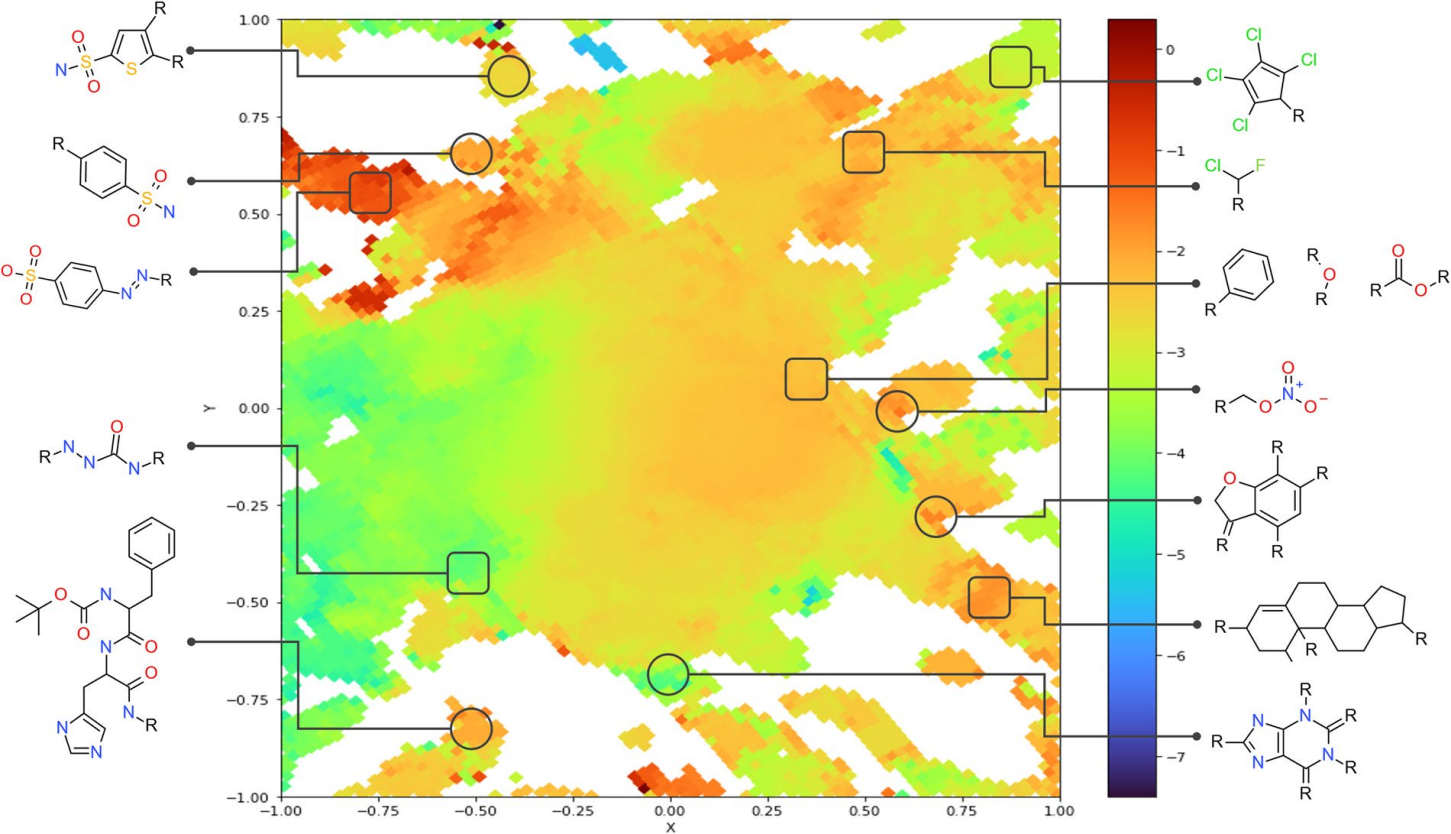
GTM landscape of the thermodynamic solubility from AqSolDBc and OChem datasets. Colors represent the experimental LogS of the aqueous solubility going from blue (poor) to red (high). Chemical space zones pertaining to specific chemotypes are highlighted. Squares and circles define areas representing respectively AqSolDBc and OChem compounds.

**Fig. 4:**
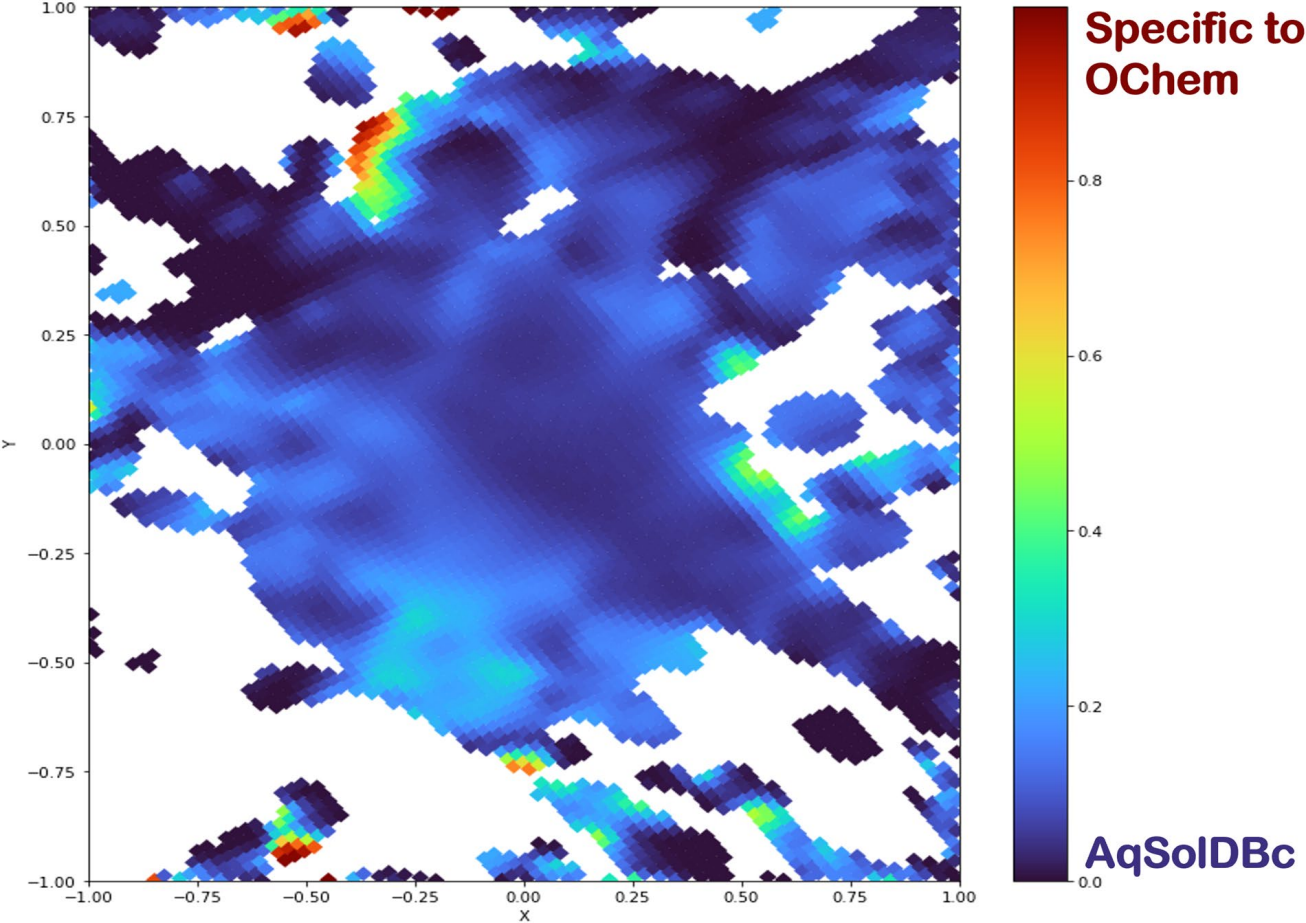
Class landscape of the test sets versus the training set, AqSolDBc. The color represents the proportion of compounds from each dataset. Blue regions are populated with structures from AqSolDBc. White spaces are unpopulated areas and red spaces are from compounds specific to OChem datasets.

The distribution of the CS over the map is shown in Fig. 2 and Fig. 3. The dense population at its center correspond to small and diverse compounds. The solubility landscape displays multiple gradients from high to poor thermodynamic solubility. The distinct chemical sets were represented on the map as class landscapes, to help comprehend how they position to one another in CS (Fig. 4). The set specific to OChem fills vacant regions of AqSolDBc CS.

### External validation

Public models were validated using public data from OChem. Priority was given to NN and models trained on AqSolDB. The validation process also involved testing the GSE (described above). We additionally trained Random Forest (RF) and MPNN (ChemProp^63^) models on AqSolDBc.

### Public data

**Fig. 5:**
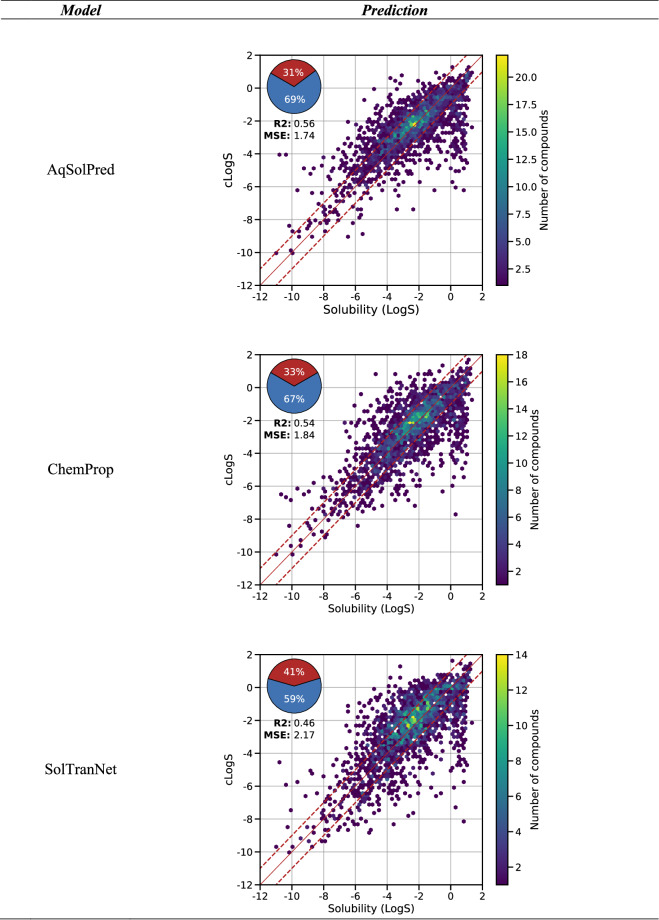
Predicted thermodynamic solubility against experimental solubility for the set specific to OChem. The red line represents a ± 1.0 log interval. The hexbins represent the density of points in the plot.

To confirm the difficulty of predicting test chemical spaces uncovered by our training set, the best performing models were applied to OChem data. We report in Fig. 5 the MSE performances over the set specific to OChem, which range from 1.74 to 2.17 log. AqSolPred shows the best performance on the two sets with an MSE of 1.74 log and R^2^ of 0.56. ChemProp presents a close MSE of 1.84 log.

### Applicability domain

The AD of a predictive model is a theoretical region of the CS covered by the model features. It delineates a region of the CS based on the similarity to the training set. Predictions on compounds in AD are considered reliable whereas out of AD they are considered uncertain. Still, few thermodynamic solubility models are delivered with an AD: Hewitt *et al*.^53^, Chevillard *et al*.^64^, Cao *et al*.^65^ and Lusci *et al*. 2013.

**Fig. 6:**
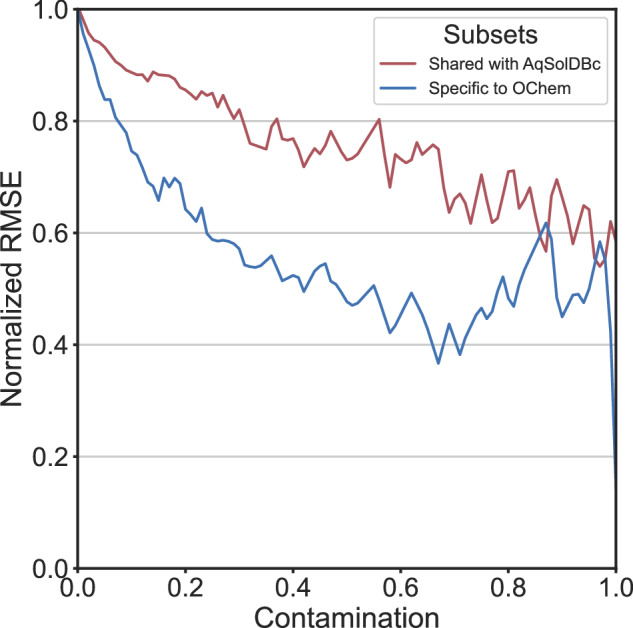
Performance of the RF model (MOE2D) using the IsolationForest Applicability Domain. Performances were computed for each increment of the contamination parameter, from 0.0 to 0.99. Normalized RMSE is the external validation RMSE at contamination X divided by the RMSE at contamination zero.

Application of an Isolation Forest based AD are resumed for RF models with MOE2D descriptors are illustrated in Fig. 6. Comparable behavior is obtained using other ML approaches. The general trend is a decrease of the RMSE as the AD coverage get more restrictive – decreasing test set coverage – with the increase of the contamination value. At some point, the test set coverage reduces too much, and the validation becomes unstable. This effect is visible on OChem data.

### Effect of the cleaning procedure from AqSolDB to AqSolDBc

To assess the impact of the cleaning procedure, several models were built on both AqSolDB and AqSolDBc datasets to observe the difference. RF models were constructed using MOE2D (n = 203) and ISIDA^66^ (8 sets, n = 284 to 22,880) descriptors. Data were split into 10 folds. For RF, nine folds were used as the training set, and one as the test set. The test set was kept consistent for all models to ensure a fair comparison. Additionally, MPNN (ChemProp) models were trained. For MPNN, eight folds were used as the training set, one as the validation set, and one as the test set. The GSE was also applied. The RMSE of MPNN, GSE, and RF are reported in Table 5. Performances over AqSolDBc should be compared to those of AqSolDB. Overall, the curation of AqSolDB resulted in a systematic improvement of the RMSE by ~0.10 log, supporting the proposed curation procedure, despite the reduced absolute training set size due to curation.

**Table 5:** Root-Mean Squared Error (RMSE) of the RF, MPNN and GSE through 10-fold CV on AqSolDBc & AqSolDB. Colors are ranged from green (low RMSE) to red (high RMSE).

Dataset	RF	RF	MPNN	GSE (Eq. 1)
	MOE2D	ISIDA	ChemProp	
AqSolDBc	0.78	0.91	0.79	1.86
AqSolDB	0.86	0.99	0.89	2.05

## Discussion

### Recommendations for the curation of solubility data

Based on this analysis, we propose a decision tree for the curation of thermodynamic solubility data (Fig. 13). It starts by a verification of the chemical structure. This can be verified using the CAS number and checking a structural database.

**Fig. 7:**
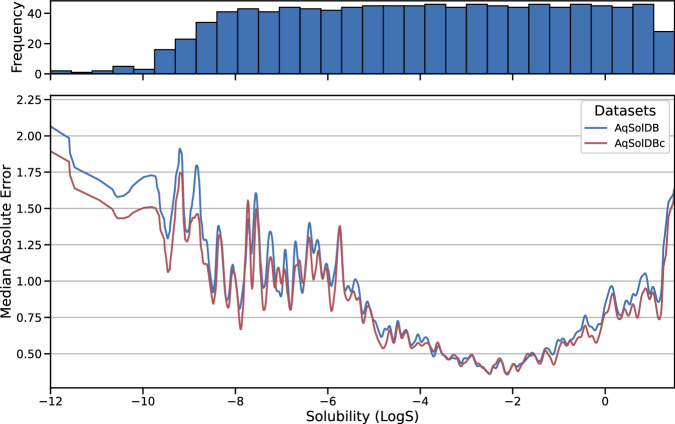
Comparison of the MAE from AqSolDB and AqSolDBc. MAE from the 10-fold CV computed over all models for AqSolDB (blue) and AqSolDBc (red) against the solubility range.

**Fig. 8:**
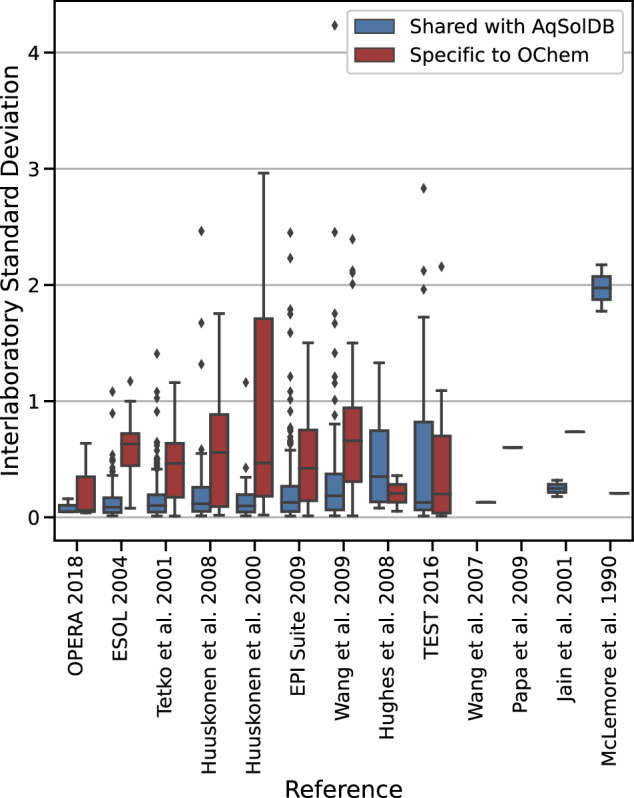
Boxplots of the experimental standard deviation (*SDi*) of compounds in the OChem database. Data shared with AqSolDB (blue) are also present in AqSolDBc, and data specific to OChem (red) are absent from AqSolDBc. Boxplots are restrained to *SDi* > 0.01 log.

**Fig. 9:**
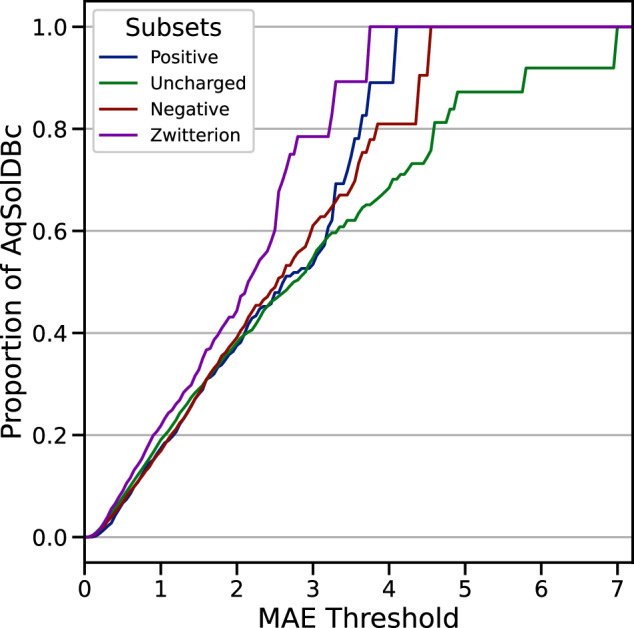
REC curve for each AqSolDBc subset corresponding to the major microspecies at pH7.0: Uncharged, Zwitterion, Negative and Positive ions. The y-axis is the proportion of AqSolDBc predicted better than a threshold MAE value on the x-axis; MAE in log from the 10-fold CV computed over all models for AqSolDBc.

**Fig. 10:**
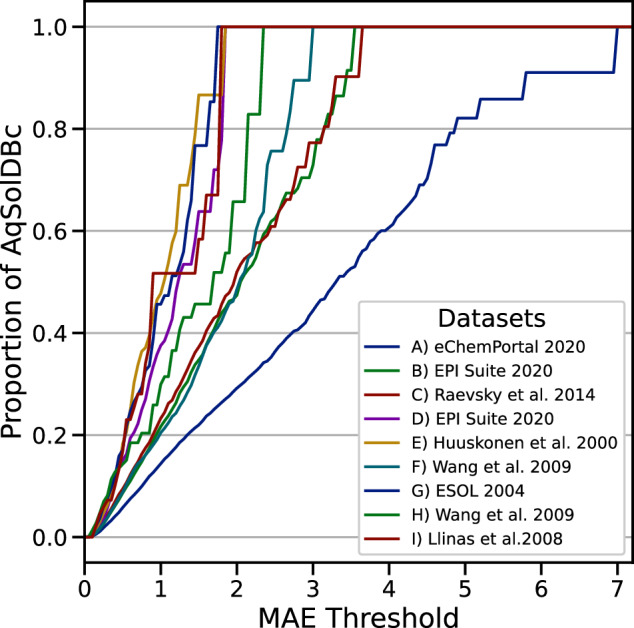
REC curve of each of the 9 AqSolDB data source. The y-axis is the proportion of AqSolDBc predicted better than a threshold MAE value on the x-axis; MAE from the 10-fold CV computed over all models for AqSolDBc.

**Fig. 11:**
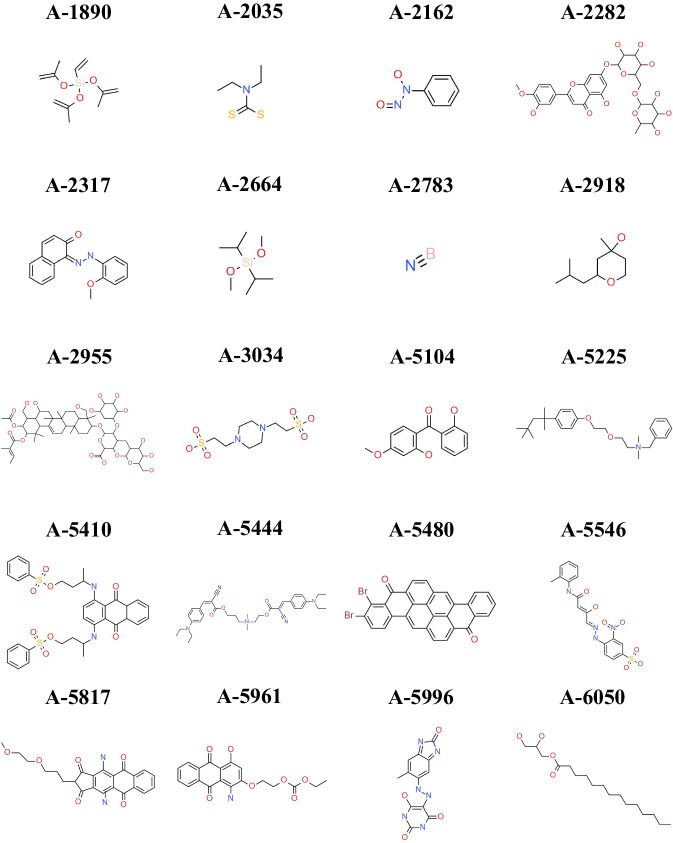
Structures and compound ID from the 20 hardest-to-predict compounds from AqSolDBc. The first letter of the ID corresponds to the source of the entry (see Fig. 10).

**Fig. 12:**
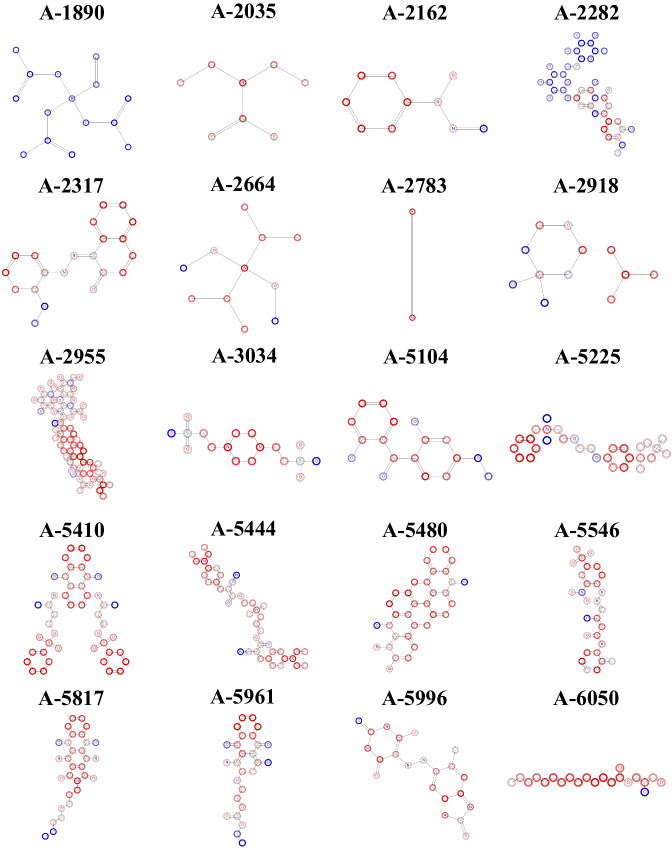
Structures and compound ID from the 20 hardest-to-predict compounds colored using ColorAtom. Coloration of compounds according to the fragment-based RF model. Red and blue regions correspond, respectively, to negative and positive contributions to LogS. Dark colors correspond to large positive or negative atomic contributions.

**Fig. 13:**
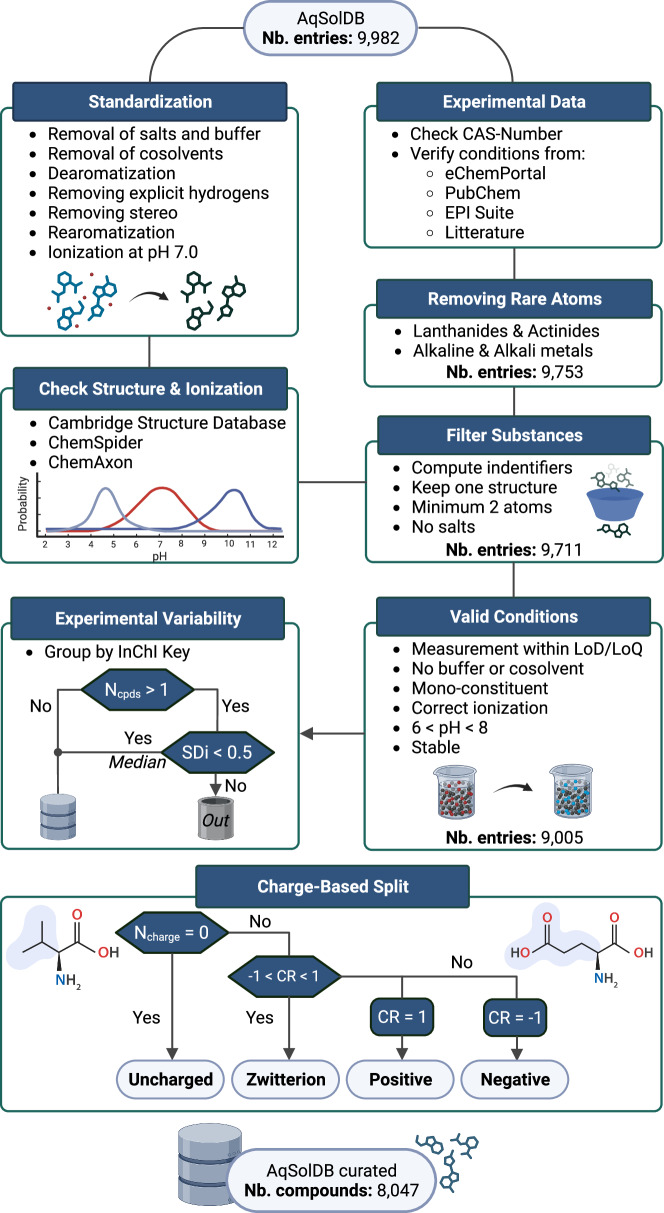
Flowchart describing the guidelines followed from compound standardization to data curation. Chemical structures are standardized and ionized using Chemaxon tools. To resolve some ambiguities the structures are verified in the ChemSpider database and in the CSD. Experimental meta-data are systematically retrieved, and the main chemical structure is extracted. The data are filtered according to the experimental conditions. When several thermodynamic solubility values are available, an entry is discarded if there is a doubt about which value to keep; otherwise, the median value is conserved.

The next step concerns the experimental protocol and its resulting *SDi* – when replica measurements are available. A crucial point to look at is the confidence of the measure. Values obtained below LOD/LOQ are subject to uncertainties and should not be used when developing regression models. One other source of variability is the substance purity as the components in solution greatly affect the measured value.

To avoid backlash, the training set should be restrained to mono-constituent substances measured at room temperature and neutral pH.

The last point revolves around the compound stability and hydrophobicity. The OECD guideline 105 recommends a water solubility cut-off of 10 mg/L for the shake-flask. Below that the column elution or slow stir should be applied, depending on the substance state, stability, and volatility. An initial idea of the method is formulated in the well-documented reviews presented by Ferguson *et al*.^67^ in 2009, and Birch Heidi *et al*. in 2019^68^. These authors introduced additional rules depending on the compound’s expected stability. Since shake-flask and column elution take few hours to days to equilibrate, the half-life cut-off is set to 24 hours. Meanwhile, the cut-off is set to 7 days for the slow-stir method as it may require weeks to equilibrate.

### External validation

Since 2017, thermodynamic solubility prediction has become a sandbox for the application of cutting-edge NN. These models present RMSE ranging from 0.35 to 1.71 log unit. Displaying good internal validation statistics may be misleading for drug designers seeking the best model. As mentioned earlier, these models often lack extensive external validation, and thus their performance should be considered with skepticism, particularly when applied to New Chemical Entities.

### Public data

To confirm the difficulty of predicting test chemical spaces uncovered by our training set, the best performing models were applied to OChem data. The relevance of previously performed external validation may be questioned. For instance, evaluating performances using sets too small, internal, or distant from a target application (i.e. pharmaceutical data) may be an issue. Validation sets, which are meant to evaluate models in the context of their specific characteristics, should be carefully chosen based on their composition, diversity, size, and quality. It is important to note that each external test set presents its own challenges due to its peculiarities (size, diversity, predominance of various chemotypes, etc.), and past success on external validation does not guarantee future performance on different test sets. Moreover, Neural Network architectures do not display any breakthrough performances. As hypothesized previously, certain prediction errors may be avoided by using an Applicability Domain (AD) with published models.

### Inter-laboratory standard deviation

The other possible source of prediction error could be the presence of poorly reproducible or variable training data. If the thermodynamic solubility is not known with sufficient accuracy or exhibits significant variability, it can introduce uncertainty into the models and distort their assessment. We analyzed the *SDi* of the OChem sets and the Median Average Error (MAE) of the set specific to OChem. The MAE is the median of the absolute difference between predictions and measurements for a given compound. Here we discuss MAE using results from a 10-fold cross-validation of ChemProp on OChem data, as a representative example model.

As OChem comprises datasets from various sources, the independent quality of each source can be investigated. To do so, the distributions of the *SDi* are confronted to the source of their entries (Fig. 8). The X-axis defines the source datasets found in OChem. To better highlight the quality of AqSolDBc, the set specific to OChem and shared with AqSolDBc are displayed as separated boxes. It is important to note that errors could be attributable to a range of factors such as measuring the solubility of the wrong compound, different solution compositions, and typos in recorded numbers or units. Furthermore, care must be taken when combining data from different temperatures or techniques to minimize the introduction of errors.

Overall, the compounds specific to OChem exhibit high *SDi* and MAE, which appear to be correlated. This suggests that the difficulties in predicting properties of compounds specific to OChem could stem from its relatively poorer data quality. The boxplots for *SDi* also show qualitative agreement. It should be noted that most compounds are well predicted, but the portion of the dataset with the highest *SDi* accounts for most of the reported error.

To summarize, these results illustrate that a decrease in measurement reliability negatively impacts the quality of models and validation.

### Impact of the data characteristics

The MAE (Median Absolute Error) was computed using the results of the 10-fold CV from all RF and MPNN models (Fig. 7) on the AqSolDBc dataset. Models trained on the AqSolDBc are overall more predictive in the high and low solubility ranges compared to those trained on AqSolDB. For compounds with thermodynamic solubility ranging from -4.0 to 0.0 log, the MAE remains below 1.0 log. It also tends to rise the further one strays from this range.

We investigated the influence of the ionization state of the principal microspecies at pH 7.0 on the error of prediction. The Charge Ratio (CR), which is the sum of charges divided by the number of charges was used to assign compounds to subsets:

- Non-Electrolytes
  - Uncharged: CR = 0
- Electrolytes
  - Zwitterion
  - Positive: CR =  + 1
  - Negative: CR = −1

Figure 9 presents the Regression Error Characteristic (REC) curves for each of these subsets obtained from the results of the 10-fold CV. They display the error tolerance expressed as MAE on the X-axis against the percentage of points predicted within the tolerance. An ideal model should be represented by a REC reaching the top left corner of the plot. It should be noted that the presence of microspecies in solution can affect the measurement, resulting in a slight difference in solubility value. Here, the defined subsets are used to highlight which compounds may be prone to these variabilities and thus give larger predictive errors. From these plots, zwitterions appear easier to predict than positively and negatively charged species. Finally, the most difficult targets are uncharged species. This is probably due to the fact that most poorly soluble species are actually uncharged, and some neutral species may be incorrectly identified as uncharged by the machine learner for rare groups.

Since AqSolDB and AqSolDBc are aggregations of public datasets, it was also possible to study the influence of data sources on the measured performances of the models (Fig. 10). The Huuskonen dataset is certainly the easiest data collection to predict. The largest errors are observed on the Raevsky, EPI Suite 2020 and, mostly eChemPortal 2020 datasets. The eChemPortal provides a lot of input data to AqSolDB, but it appears that they might be a large source of erroneous entries. Therefore, the eChemPortal dataset requires a closer look which is out of the scope of this study.

### Hard-to-predict compounds

Finally, the information concerning the 20 hardest-to-predict compounds (having the largest MAE) from AqSolDBc are reported in Table 6 and Fig. 11. Most of them are hydrophobic compounds from eChemPortal and measured using the shake-flask method. However, the OECD 105 advises to use the column elution with poorly soluble molecules. The usual lack of confidence over poorly soluble substance can be partially explained by the non-respect of the OECD.

**Table 6:** Information concerning the experimental conditions of the 20 hardest-to-predict compounds from AqSolDBc.

ID	CAS	LogS	Remark	Method
A-5961	40530-60-7	−9.22	N.C	Flask
A-2317	1229-55-6	−8.93	Valid	N.S
A-5817	65059-45-2	−8.27	N.C	Flask
A-5546	CID: 83010	−7.74	N.C	N.S
A-2282	520-27-4	−7.51	Valid	Flask
A-5104	131-53-3	−7.27	Valid	Flask
A-5996	72102-84-2	−6.49	Below LOD	Flask
A-2783	10043-11-5	−6.39	Valid	N.S
A-2664	18230-61-0	−6.25	N.C	N.S
A-2162	15305-07-4	−6.19	Valid	Column elution
A-2035	14324-55-1	−5.53	Unstable	Column elution
A-5480	1324-35-2	−4.45	N.C	Flask
A-3034	10010-67-0	−2.75	Self-buffering	N.S
A-2955	26339-90-2	−1.10	Valid	N.S
A-5444	78181-99-4	−0.80	Unstable	N.S
A-5410	70900-27-5	−0.44	Valid	Flask
A-5225	121-54-0	0.07	Valid	Flask
A-1890	15332-99-7	0.65	Unstable	QSAR
A-2918	63500-71-0	2.14	N.C	N.S

The 20 hardest-to-predict compounds display the highest MAE over all models. Remarks accounting for non-valid conditions to our guidelines are specified. The first letter of the ID corresponds to the source of the entry (see Fig. 10). N.C: Non-Conclusive, N.S: Not Specified.

### Interpretation of the model

To evaluate the contribution of each atom into the modelled solubility, we employed ColorAtom^69,70^. This interface employed our RF model based on ISIDA fragment descriptors to produce chemical structures where each atom bears an atomic contribution of the value calculated by the model. The 20 hardest-to-predict compounds were passed on ColorAtom. Their colored structures are reported Fig. 12. As expected, the polar parts of the molecules are usually colored in blue (high solubilization) whereas aromatic and aliphatic moieties are in red (poor solubilization).

### Key results

In our study, we conducted an extensive analysis of thermodynamic solubility using two datasets: AqSolDBc and OChem. Our findings underscored the complexities and challenges of solubility prediction, but also highlighted potential strategies for improvement.

The mapping of chemical space revealed a diverse range of the solubility subspaces, highlighting the value of using diverse and complementary datasets. Despite the diversity of data, external validation revealed that all models struggled. This finding underscored the importance of model refinement and the need to consider the applicability domain when applying models to novel data. Moreover, the curation of AqSolDB into AqSolDBc significantly improved the RMSE, showing that data cleaning procedures can substantially enhance prediction accuracy.

Our study also revealed that inter-laboratory variability and the source of data can significantly influence model performance. This highlights the importance of measurement reliability and stringent data validation procedures, raising questions about the quality of datasets like eChemPortal.

Our study corroborates the findings of Lowe *et al*.^29^, emphasizing the complexity and challenges in solubility prediction across diverse chemical spaces. We found that RF models provide a balanced and interpretable framework. The model’s interpretation underscored the essential role of fragment-based modeling approaches in elucidating the underlying mechanisms of the predictions. These insights underline the importance of the application of OECD^68^ principles for enhancing predictive accuracy and interpretability. Additionally, we investigated the 20 hardest-to-predict compounds, most of which were hydrophobic and measured using unsuitable methods. This underscored the need of carefully selecting entries based on their experimental procedure, to which we answered by delivering a decision tree for the curation of solubility data.

Overall, our findings indicate that while advancements have been made in the field of solubility prediction, challenges remain. These insights offer valuable guidance for future research and model refinement.

### Summary

Published solubility models often display attractive performances. However, these same models very often fail in prospective predictions. This work aimed at clarifying the reasons for these repeated failures.

First, we compiled a comprehensive list of solubility datasets and highlighted their interconnections. It appears that some data sources are overlooked and others frequently aggregated.

Second, we observed that the use of sophisticated neural network architectures did not lead to any breakthrough, although major scientific discussions were triggered by both solubility challenges 1 and 2.

Third, when applied to an external public dataset, all models performed poorly. This is probably due to an applicability domain issue.

Fourth, we conducted a thorough reevaluation of the popular AqSolDB dataset to address potential inconsistencies and improve its quality. Our analysis led to the creation of a new version of the dataset, which exhibits improved internal consistency by ensuring that the data points are more reliable and better adhere to the principles of solubility prediction. This revised dataset allows for a more accurate assessment of factors that impact the performance of solubility prediction models, ultimately leading to better model development and evaluation. This allowed us to observe the influence of factors impacting the performances of the models: the laboratory standard deviation, the ionic state of the solute, and the source of the solubility data. It appears that the eChemPortal probably contains some corrupted data and requires careful data cleaning.

Lastly, we provide a thoroughly curated version of AqSolDB called AqSolDBc, obtained following a decision tree based on experimental conditions. With these rules, we hope to offer a correct way to curate aqueous solubility data. This set was used to train RF and MPNN models for solubility prediction and IsolationForest models for Applicability Domain. Models trained on public data, applied during this project are publicly available (https://chematlas.chimie.unistra.fr/WebTools/predictor_solubility.php).

## Methods

### Data curation

For these approaches to produce accurate predictions over a vast CS, a high quality and diversified training set is a must. However, preserving accurate measurements necessitates accounting for experimental variability, often evaluated with the *SDi*. Experimental thermodynamic solubility data can have inaccuracies up to 1.5 log, according to John C. Dearden^71^. Additionally, Llinas *et al*. reported that measurements between laboratories may vary by 0.5 to 0.6 log. Poor reproducibility can be the consequence of unintentional mistakes brought on by combining entries with heterogenous conditions, or of poor quality^52^.

**Fig. 14:**
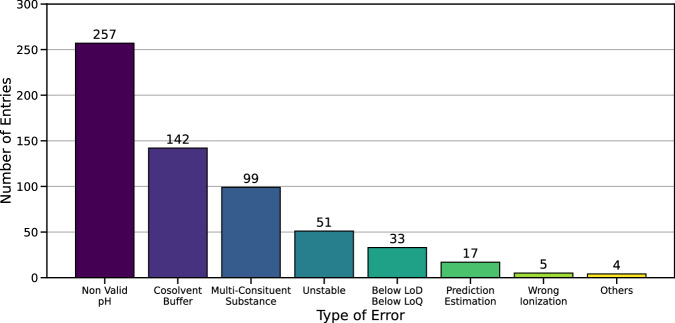
Number of non-valid entries in AqSolDB identified with the help of the meta-data of measurement.

In the following, we propose a guideline for the improvement of thermodynamic solubility data set quality, which we applied to AqSolDB. This dataset, aggregated by Sorkun *et al*.^25^ in 2020, was chosen for its size, diversity, and well referenced entries. To curate AqSolDB and obtain an experimentally homogenous library, we followed the flowchart illustrated in Fig. 13. Chemaxon’s JChem^72^ software was employed for structural database standardization. In case of ambiguities, chemical structures were verified in ChemSpider^73^ to benefit from its crowd sourced annotations. When possible, these structures were also searched in the CSD where the values of bond lengths, angles and torsions help to disambiguate the nature of chemical functions. CAS numbers were verified using SciFinder^74^ before using them to retrieve manually described experimental conditions from eChemPortal^75^, EPI Suite^20^, and PubChem^76^ if available. Overall, 608 entries containing partial records on start and final pH, measurement limitation, composition, origin, stability, or cosolvents were reported (Fig. 14). The forementioned experimental conditions and their importance to modelers are discussed.

#### pH sensitive species

The thermodynamic solubility of ionizable compounds strongly depends on the pH and the presence of buffer or ions. These factors can influence the microspecies equilibrium by interacting with the solute. For instance, the counter-ion effect can increase, or decrease this solubility. Therefore, several control steps are recommended:

- Verifying the validity of the reported salt structure using its CAS number. This is manageable using the SciFinder^74^ database and verifying when possible, in the Cambridge Structural Database^77^ (CSD).
- Selecting measurements without buffer, added acids/bases, cosolvents and surfactants.
- Restraining the data to entries reporting a final pH = 7 ± 1.

Ionized compounds obtained through standardization should correspond to the major microspecies in solution. The microspecies distributions have been obtained using ChemAxon pKa Plugin^44^. Compounds presenting too many microspecies (more than 4) and those with uncertain major microspecies at pH 7.0 have been excluded, because we could not decide which structure to use for modeling.

Overall, 399 entries from AqSolDB obtained in the presence of buffer, cosolvent, or undesirable pH were excluded. Five entries were also deemed uncertain for having ionized structures different from the major microspecies or poor microspecies distribution.

#### Substance composition

Water solubility is a property of pure compounds. However, it is sometimes reported for substances. Pure compounds solubilities cannot be considered together with complex substances solubilities. The European Chemical Agency^38^ describes three types of substance:

- UVCB (Unknown or Variable composition, Complex reaction productions or biological materials), contain several chemicals without a complete understanding of their identity. Their composition is variable and often unknown. They usually originate from industrial processes or biological extracts.
- Multi-constituent, account for a mix of known chemicals and impurities. Reported ingredients should represent 10% to 80% of the substance.
- Mono-constituent refers to a solute that only contains one major component with up to 20% impurity. However, this level of purity is still high and can have a significant impact on solubility, bioactivity, and other important factors. It should be noted that such a high level of impurities can negatively affect the results and should be taken into consideration during their interpretation.

Ninety-nine entries from AqSolDB were found and eliminated for being UVCB, or multi-constituent substances (Fig. 14).

### Unstable species

Chemical stability is related to the degradation processes. In solution, the compound can be subject to hydrolysis, hydration (R-(C=O)-R’ → R-C(OH)_2_-R’), photolysis, oxidation, biodegradation, and polymerization. These are generally dependent on the pH and temperature. The hydrolysis represents the most difficult ones to avoid during experimentation. Solubility test systems can limit photolysis by using amber glass bottles, aluminum or be done in the dark. Oxidation can be limited by working under anaerobic conditions, through nitrogen or argon flushing or by limiting the air headspace. Chemicals for which hydrolysis rapidly occur should be excluded to avoid measurements altered by reaction products. Care should be taken with compounds containing reactive functional groups such as mono- and poly- halogenated aliphatic (alkyl halides), epoxides, organophosphorus esters, carboxylic acid esters, carbamates, nitriles, organometallic, and peroxides. The Degradation Time (DT50) can be used to investigate the compounds stability. The DT50 is the period after which half of the original amount of chemical is degraded. Hydrophilic compounds with a DT50 lower than 24 hours and hydrophobic with a DT50 lower than 7 days should be discarded^68^. We identified 52 such entries in AqSolDB. Reversible reactions with water, such as hydration of activated aldehydes or internal hemiacetal formation in sugars are not *de facto* signaling compound instability but are sources of prediction error because the actual “solute” structures differ from the input standard form of the molecule.

#### Other errors

We identified 17 suspicious entries in AqSolDB resulted from either averaging measurement of similar chemicals or predictions with ML methods. In our opinion, such values should not be used for model building. Lastly, the experimental procedure may be biased. For example, two entries were discarded because the calibration of instruments was performed under different conditions than used to run the test samples.

#### Duplicate measurements

A common outcome of datasets aggregation is the occurrence of duplicated measurements. Managing them is a chance to investigate uncertainties. However, it is desirable to maintain one value per structure, preferably the median. This only make sense when reported values are relatively close. When there are only two very different values, or there are two or three clusters of different values associated to compounds with the same InChI Key, the median or average value becomes meaningless. Such cases are filtered out by a *SDi* > 0.5 log threshold.

The result of this process to the AqSolDB is labeled AqSolDBc in the following.

### Test Set Curation

Based on the number of entries, OChem represents the largest thermodynamic solubility repository. More than half of them are from AqSolDB, EPI Suite, VEGA^78^, TEST^79^ and OPERA^80^. Following standardization, 7,463 unique structures remained, with values ranging from –13.17 to 1.70 log units. Out of these, 70% are found to overlap with AqSolDBc. To assess the model’s performance on both overlapping and unique compounds from the OChem dataset, it was divided into two subsets: a set shared with AqSolDBc containing 5,212 compounds and a set specific to OChem with 2,251 compounds, which were harder to predict.

### Chemical space maps

The various compound sets were compared using Generative Topographic Mapping (GTM). The GTM method inserts a manifold into a N-dimensional molecular descriptor space populated by a set of representative chemical structures. By shifting the centers of Radial Basis Functions, the technique maximizes the log likelihood (LLh) while fitting the manifold to data. Subsequently, the data points are projected onto the manifold before unbending it. A vector of normalized probabilities (responsibilities), computed on the nodes of a grid over the manifold, is used to represent each compound in the latent space. The complete data set can therefore be described as a vector of cumulative responsibilities which is figured as a map and termed as a *landscape*.

Here, a combined dataset composed of 4,463 unique structures was created from AqSolDBc and OChem. ISIDA descriptors were employed for GTM training, as previous studies demonstrated their comprehensive coverage of the relevant chemical space and their ability to effectively represent molecular structures^81^. The descriptor space includes descriptors related to aromaticity as well as ISIDA counts of sequences and fragments from 2 to 3 atoms, representing a total of 6,121 distinct fragments (Nomenclature: IIAB(2-3)_CI)^82^. The GTM manifold was trained using 100 iterations before being resampled to obtain a map of 8,000 nodes. The map is colored based on property and class values, which subsequently generate property and class landscapes for data set comparisons. To achieve this, the responsibility-weighted mean of the class labels/property values of resident objects is obtained from each node’s mean class/property value^83^.

### External validation

Public models were validated using public data from OChem. Priority was given to NN and models trained on AqSolDB. The validation process also involved testing the GSE (described above).

- AqSolPred is a consensus predictor based on 3 models originally trained with a version of AqSolDB depleted of eChemPortal and EPI Suite subsets. Authors used 123 2D descriptors in NN, RF and XGBoost methods. Their consensus model scored a RMSE of 0.35 log on the Huuskonen benchmark dataset.
- SolTranNet also uses the SMILES representation. It is built upon a molecule attention transformer (MAT) architecture. It applies self-attention to molecular graph representation, where each node is characterized by a feature vector which is then combined with the adjacency and distance matrices of the molecule. The distance matrix is built on a minimized 3D model of the molecule.

For training QSAR models on AqSolDBc we used Random Forest (RF) and MPNN (ChemProp^63^). The RF is from scikit-learn^84^ implementation with MOE2D^85^ descriptors excluding LogS and (number of descriptors = 203) to limit the usage of predicted properties as descriptors. Using other software suite such as ISIDA led to similar results. We also used OChem models ($LogPo/w$: *ALOGPS 2.1*, 2016; MP: *Best estate*, 2015) to predict $LogPo/w$ and MP and used the computed values as input to the GSE. The ChemProp MPNN model is a Directed Message Passing Neural Network (D-MPNN) renowned for producing reliable predictive models of chemical properties. Finally, ChemProp was used alone and in consensus with AqSolPred.

The consensus prediction was conducted to improve the applicability of AqSolPred as it was trained with a version of AqSolDB lacking eChemPortal and EPI Suite. Following the guidelines shared by the authors, models were used as intended: the performances announced were retrieved. Models were applied to 7,463 compounds from OChem.

### Applicability domain

We used Isolation Forest^86^ models as AD to verify our hypothesis. The Isolation Forest method constructs an ensemble of trees for a given dataset. During the tree-building process, each tree is grown by recursively selecting a random feature and a random split value between the minimum and maximum values of the selected feature to partition the observations. Instances with short average path lengths within the trees are identified as outliers. The essence of the Isolation Forest algorithm lies in this random partitioning to identify outliers. The IsolationForest models were trained with AqSolDBc (MOE2D descriptors, n = 203) using scikit-learn^84^ with an increasing contamination parameter, from 0.0 to 0.99.

**Fig. 15:**
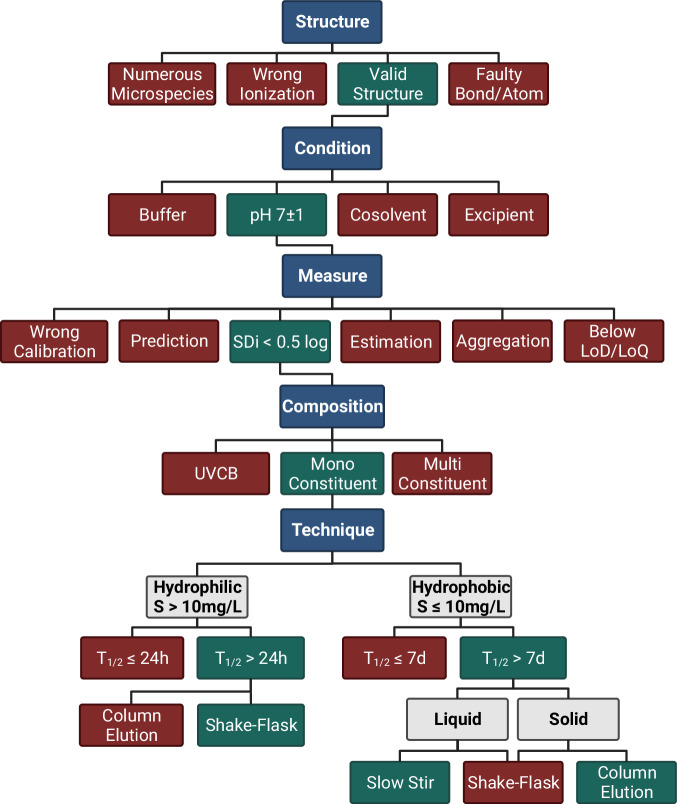
Decision tree proposed for the curation of thermodynamic solubility data. Red nodes define non-valid conditions or chemical states, and green nodes account for correct entries.

The contamination parameter defines the expected proportion of outliers within the training set and is used by the Isolation Forest as a threshold to discriminate outliers from inliers. In other words, a contamination of 0 corresponds to a 100% coverage of the applicability domain (no molecule rejected) and a contamination of 1 corresponds to a 0% coverage of the applicability domain (all molecule rejected). OChem’s set was applied to these models. The RMSE from the compounds within the AD was computed for each incrementation of the contamination Fig. 15.

## Data Availability

The authors declare that the data supporting the findings of this study are available free of charge^6^. The repository features multiple datasets that have been curated for this research. The repository contains the following files: File **AqSolDBc.csv** Curated data from the AqSolDB. The available columns are: • *ID* Compound ID (string) • *InChI* InChI code of the chemical structure (string) • *Solubility* Mole/L logarithm of the thermodynamic solubility in water at pH 7 (+/−1) at ~300 K (float) • *SMILEScurated* Curated SMILES code of the chemical structure (string) • *SD* Standard laboratory Deviation, default value: −1 (float) • *Group* Data quality label imported from AqSolDB (string) • *Dataset* Source of the data point (string) • *Composition* Purity of the substance: mono-constituent, multi-constituent, UVCB (Categorical) • *Error* Identifier error on the data point, default value: None (String) • *Charge* Estimated formal charge of the compound at pH 7: Positive, Negative, Zwiterion, Uncharged (Categorical) File **OChemUnseen.csv** Solubility data from OChem, curated and orthogonal to AqSolDB. The available columns are: • *SMILES* Curated SMILES code of the chemical structure (string) •*LogS* Mole/L logarithm of the thermodynamic solubility in water at pH 7 ( + /−1) (float) File **OChemOverlapping.csv** Solubility data from OChem, curated; chemical structures are also present inside AqSolDB. The available columns are: • *SMILES* Curated SMILES code of the chemical structure (string) • *LogS* Mole/L logarithm of the thermodynamic solubility in water at pH 7 ( + /−1) (float) File **OChemCurated.csv** Solubility data from OChem, curated. The available columns are: • *ID* Compound ID (string) • *Name* Compound name (string) • *SMILES* Curated SMILES code of the chemical structure (string) • *SDi* Standard laboratory Deviation, default value: −1 (float) • *Reference* Unformated bibliographic reference which the data point is originating from (string) • *LogS* Mole/L logarithm of the thermodynamic solubility in water at pH 7 ( + /−1) (float) • *EXTERNALID* Compound ID as appearing in its data source, default value: None (string) • *CASRN* CAS number of the compound, default value: None (string) • *ARTICLEID* Source ID linked to the column Reference (string) • *Temperature* Temperature of the measure, in K (float)

## Code availability

No custom code has been used.

## References

[1] Kennedy T (1997). Managing the drug discovery/development interface. Drug Discov. Today.

[2] Kola I, Landis J (2004). Can the pharmaceutical industry reduce attrition rates?. Nat. Rev. Drug Discov..

[3] Millard J, Alvarez-Núñez F, Yalkowsky S (2002). Solubilization by cosolvents. Establishing useful constants for the log-linear model. Int. J. Pharm..

[4] Jouyban A, Abolghassemi Fakhree MA (2008). Solubility prediction methods for drug/drug like molecules. Recent Pat. Chem. Eng..

[5] van de Waterbeemd H (2009). Improving compound quality through in vitro and in silico physicochemical profiling. Chem. Biodivers..

[6] Llompart P (2023). Recherche Data Gouv.

[7] Wang J, Hou T (2011). Recent advances on aqueous solubility prediction. Comb. Chem. High Throughput Screen..

[8] Elder DP, Holm R, Diego HL (2013). Use of pharmaceutical salts and cocrystals to address the issue of poor solubility. Int. J. Pharm..

[9] Saal C, Petereit AC (2012). Optimizing solubility: Kinetic versus thermodynamic solubility temptations and risks. Eur. J. Pharm. Sci..

[10] Wang J (2007). Development of reliable aqueous solubility models and their application in druglike analysis. J. Chem. Inf. Model..

[11] Johnson SR, Zheng W (2006). Recent progress in the computational prediction of aqueous solubility and absorption. AAPS J..

[12] Delaney JS (2005). Predicting aqueous solubility from structure. Drug Discov. Today.

[13] OECD. Test No. 105: Water Solubility. *OECD Guidelines for the Testing of Chemicals, Section 1* https://read.oecd-ilibrary.org/environment/test-no-105-water-solubility_9789264069589-en (1995).

[14] Llinàs A, Glen RC, Goodman JM (2008). Solubility Challenge: Can You Predict Solubilities of 32 Molecules Using a Database of 100 Reliable Measurements?. J. Chem. Inf. Model..

[15] Stuart M, Box K (2005). Chasing Equilibrium:  Measuring the Intrinsic Solubility of Weak Acids and Bases. Anal. Chem..

[16] Huuskonen J, Rantanen J, Livingstone D (2000). Prediction of aqueous solubility for a diverse set of organic compounds based on atom-type electrotopological state indices. Eur. J. Med. Chem..

[17] Yalkowsky, RM & Dannenfleser, SH. Aquasol database of aqueous solubility. Version 5. https://hero.epa.gov/hero/index.cfm/reference/details/reference_id/5348039 (2009).

[18] Bloch, D. Computer Software Review. Review of PHYSPROP Database (Version 1.0). *ACS Publications* https://pubs.acs.org/doi/pdf/10.1021/ci00024a602 (2004) 10.1021/ci00024a602.

[19] Dalanay JS (2004). ESOL:  Estimating Aqueous Solubility Directly from Molecular Structure. J. Chem. Inf. Comput. Sci..

[20] US EPA. EPI Suite. https://www.epa.gov/tsca-screening-tools/epi-suitetm-estimation-program-interface

[21] Wang J, Hou T, Xu X (2009). Aqueous Solubility Prediction Based on Weighted Atom Type Counts and Solvent Accessible Surface Areas. J. Chem. Inf. Model..

[22] Boobier S, Hose DRJ, Blacker AJ, Nguyen BN (2020). Machine learning with physicochemical relationships: solubility prediction in organic solvents and water. Nat. Commun..

[23] Tetko IV, Tanchuk VY, Kasheva TN, Villa AEP (2001). Estimation of Aqueous Solubility of Chemical Compounds Using E-State Indices. J. Chem. Inf. Comput. Sci..

[24] Avdeef A (2020). Prediction of aqueous intrinsic solubility of druglike molecules using Random Forest regression trained with Wiki-pS0 database. ADMET DMPK.

[25] Sorkun MC, Khetan A, Er S (2019). AqSolDB, a curated reference set of aqueous solubility and 2D descriptors for a diverse set of compounds. Sci. Data.

[26] Sushko I (2011). Online chemical modeling environment (OCHEM): web platform for data storage, model development and publishing of chemical information. J. Comput. Aided Mol. Des..

[27] Panapitiya G (2022). Evaluation of Deep Learning Architectures for Aqueous Solubility Prediction. ACS Omega.

[28] Wiercioch M, Kirchmair J (2021). Dealing with a data-limited regime: Combining transfer learning and transformer attention mechanism to increase aqueous solubility prediction performance. Artif. Intell. Life Sci..

[29] Lowe CN (2023). Transparency in Modeling through Careful Application of OECD’s QSAR/QSPR Principles via a Curated Water Solubility Data Set. Chem. Res. Toxicol..

[30] Francoeur PG, Koes DR (2021). SolTranNet-A Machine Learning Tool for Fast Aqueous Solubility Prediction. J. Chem. Inf. Model..

[31] Sluga, J., Venko, K., Drgan, V. & Novič, M. QSPR Models for Prediction of Aqueous Solubility: Exploring the Potency of Randić-type Indices. *Croat. Chem. Acta* **93** (2020).

[32] Meng J (2022). Boosting the predictive performance with aqueous solubility dataset curation. Sci. Data.

[33] Lee S (2022). Novel Solubility Prediction Models: Molecular Fingerprints and Physicochemical Features vs Graph Convolutional Neural Networks. ACS Omega.

[34] Schrödinger. QikProp. (2015).

[35] United States National Library of Medicine. ChemIDplus advanced. https://pubchem.ncbi.nlm.nih.gov/source/ChemIDplus (2011).

[36] Kühne R, Ebert R-U, Kleint F, Schmidt G, Schüürmann G (1995). Group contribution methods to estimate water solubility of organic chemicals. Chemosphere.

[37] OECD. eChemPortal: The Global Portal to Information on Chemical Substances, https://www.echemportal.org/echemportal/ (2023).

[38] European Chemicals Agency. ECHA. https://echa.europa.eu/fr/ (2023).

[39] Irmann F (1965). Eine einfache Korrelation zwischen Wasserlöslichkeit und Struktur von Kohlenwasserstoffen und Halogenkohlenwasserstoffen. Chem. Ing. Tech..

[40] Hansch C, Quinlan JE, Lawrence GL (1968). Linear free-energy relationship between partition coefficients and the aqueous solubility of organic liquids. J. Org. Chem..

[41] Yalkowsky SH, Valvani SC (1980). Solubility and partitioning I: Solubility of nonelectrolytes in water. J. Pharm. Sci..

[42] Ran Y, Yalkowsky SH (2001). Prediction of drug solubility by the general solubility equation (GSE). J. Chem. Inf. Comput. Sci..

[43] Hansen NT, Kouskoumvekaki I, Jørgensen FS, Brunak S, Jónsdóttir SÓ (2006). Prediction of pH-Dependent Aqueous Solubility of Druglike Molecules. J. Chem. Inf. Model..

[44] ChemAxon. Marvin. https://chemaxon.com/products/marvin (2023).

[45] Johnson SR, Chen X-Q, Murphy D, Gudmundsson O (2007). A Computational Model for the Prediction of Aqueous Solubility That Includes Crystal Packing, Intrinsic Solubility, and Ionization Effects. Mol. Pharm..

[46] Hopfinger, A. J., Esposito, E. X., Llinàs, A., Glen, R. C. & Goodman, J. M. Findings of the Challenge To Predict Aqueous Solubility. *ACS Publications* https://pubs.acs.org/doi/pdf/10.1021/ci800436c (2008). 10.1021/ci800436c 19117422

[47] Lusci A, Pollastri G, Baldi P (2013). Deep architectures and deep learning in chemoinformatics: the prediction of aqueous solubility for drug-like molecules. J. Chem. Inf. Model..

[48] Huuskonen J, Livingstone DJ, Manallack DT (2008). Prediction of drug solubility from molecular structure using a drug-like training set. SAR QSAR Environ. Res..

[49] Zhou D, Alelyunas Y, Liu R (2008). Scores of Extended Connectivity Fingerprint as Descriptors in QSPR Study of Melting Point and Aqueous Solubility. J. Chem. Inf. Model..

[50] Erić S, Kalinić M, Popović A, Zloh M, Kuzmanovski I (2012). Prediction of aqueous solubility of drug-like molecules using a novel algorithm for automatic adjustment of relative importance of descriptors implemented in counter-propagation artificial neural networks. Int. J. Pharm..

[51] Llinas A, Avdeef A (2019). Solubility Challenge Revisited after Ten Years, with Multilab Shake-Flask Data, Using Tight (SD ∼ 0.17 log) and Loose (SD ∼ 0.62 log) Test Sets. J. Chem. Inf. Model..

[52] Llinas A, Oprisiu I, Avdeef A (2020). Findings of the Second Challenge to Predict Aqueous Solubility. J. Chem. Inf. Model..

[53] Hewitt M (2009). In silico prediction of aqueous solubility: the solubility challenge. J. Chem. Inf. Model..

[54] Goh, G. B., Hodas, N., Siegel, C. & Vishnu, A. SMILES2vec: Predicting Chemical Properties from Text Representations. Preprint at arXiv:1712.02034 (2018).

[55] Cui, Q. *et al*. Improved Prediction of Aqueous Solubility of Novel Compounds by Going Deeper With Deep Learning. *Front. Oncol*. **10** (2020). 10.3389/fonc.2020.00121 PMC7026387 32117768

[56] Maziarka, Ł. et al. *Molecule Attention Transformer*. (2020).

[57] Lovrić M (2021). Machine learning in prediction of intrinsic aqueous solubility of drug-like compounds: Generalization, complexity, or predictive ability?. J. Chemom..

[58] Kohavi, R. & Wolpert, D. H. in *International Conference on Machine Learning* Bias Plus Variance Decomposition for Zero-One Loss Function (1996).

[59] Dwork C (2015). The reusable holdout: Preserving validity in adaptive data analysis. Science.

[60] Breiman L, Spector P (1992). Submodel Selection and Evaluation in Regression. The X-Random Case. Int. Stat. Rev. Rev. Int. Stat..

[61] Rao, R. B., Fung, G. & Rosales, R. in *Proceedings of the 2008 SIAM International Conference on Data Mining (SDM)* On the Dangers of Cross-Validation. An Experimental Evaluation. 588–596 (Society for Industrial and Applied Mathematics, 2008).

[62] Rytting, E., Lentz, K. A., Chen, X. Q., Qian, F. & Vakatesh S. Aqueous and cosolvent solubility data for drug-like organic compounds. *AAPS J*. **7**, E78–105, 10.1208/aapsj070110 (2005). 10.1208/aapsj070110 PMC2751500 16146352

[63] Heid, E. *et al*. Chemprop: A Machine Learning Package for Chemical Property Prediction. *J. Chem. Inf. Model*. **64**, 9–17, 10.1021/acs.jcim.3c01250 (2024). 10.1021/acs.jcim.3c01250 PMC10777403 38147829

[64] Chevillard F (2012). In Silico Prediction of Aqueous Solubility: A Multimodel Protocol Based on Chemical Similarity. Mol. Pharm..

[65] Cao D-S, Xu Q-S, Liang Y-Z, Chen X, Li H-D (2010). Prediction of aqueous solubility of druglike organic compounds using partial least squares, back‐propagation network and support vector machine. J. Chemometrics..

[66] Ruggiu F, Marcou G, Varnek A, Horvath D (2010). ISIDA Property-Labelled Fragment Descriptors. Mol. Inform..

[67] Ferguson AL, Debenedetti PG, Panagiotopoulos AZ (2009). Solubility and Molecular Conformations of n-Alkane Chains in Water. J. Phys. Chem. B.

[68] Birch H, Redman AD, Letinski DJ, Lyon DY, Mayer P (2019). Determining the water solubility of difficult-to-test substances: A tutorial review. Anal. Chim. Acta.

[69] Marcou, G., Horvath, D. & Solov, V. Interpretability of SAR/QSAR Models of any Complexity by Atomic Contributions. *Mol Inf*. 10.1002/minf.201100136 27477814

[70] OECD. Principles For The Validation, For Regulatory Purposes, of QSAR models. https://www2.oecd.org/chemicalsafety/risk-assessment/37849783.pdf (2004).

[71] Dearden JC (2006). In silico prediction of aqueous solubility. Expert Opin. Drug Discov..

[72] ChemAxon. JChem Base, version 22.19.0 (2022).

[73] Ayers, M. ChemSpider: The Free Chemical Database. *Royal Society of Chemistry* https://www.chemspider.com (2023)

[74] CAS. SciFinder. https://scifinder.cas.org (2023).

[75] OECD, eChemPortal, https://www.echemportal.org/echemportal/.

[76] Kim S (2021). PubChem in 2021: new data content and improved web interfaces. Nucleic Acids Res..

[77] Groom CR, Bruno IJ, Lightfoot MP, Ward SC (2016). The Cambridge Structural Database. Acta Crystallogr. Sect. B Struct. Sci. Cryst. Eng. Mater..

[78] Pedretti A, Mazzolari A, Gervasoni S, Fumagalli L, Vistoli G (2021). The VEGA suite of programs: an versatile platform for cheminformatics and drug design projects. Bioinformatics..

[79] US EPA. User’s Guide for T.E.S.T. (version 4.2) (Toxicity Estimation Software Tool) A Program to Estimate Toxicity from Molecular Structure. https://www.epa.gov/chemical-research/users-guide-test-version-42-toxicity-estimation-software-tool-program-estimate (2016).

[80] Mansouri K, Grulke CM, Judson RS, Williams AJ (2018). OPERA models for predicting physicochemical properties and environmental fate endpoints. J. Cheminformatics.

[81] Lin A (2018). Mapping of the Available Chemical Space versus the Chemical Universe of Lead-Like Compounds. ChemMedChem.

[82] Bonachera, F. Isida/fragmentor 2017 user guide. 25.

[83] Gaspar HA, Baskin II, Marcou G, Horvath D, Varnek A (2015). GTM-Based QSAR Models and Their Applicability Domains. Mol. Inform..

[84] Pedregosa, F. *et al* Scikit-learn: Machine Learning in Python. *Journal of Machine Learning Research* 2825–2830 (2011).

[85] Chemical Computing Group ULC. Molecular Operating Environment (MOE). (2022).

[86] Liu, F. T., Ting, K. M. & Zhou, Z.-H. in *2008 Eighth IEEE International Conference on Data Mining*. Isolation Forest. 413–422 (2008).

[87] Huuskonen J, Salo M, Taskinen J (1997). Neural Network Modeling for Estimation of the Aqueous Solubility of Structurally Related Drugs. J. Pharm. Sci..

[88] Bruneau P (2001). Search for Predictive Generic Model of Aqueous Solubility Using Bayesian Neural Nets. J. Chem. Inf. Comput. Sci..

[89] Liu R, So S-S (2001). Development of Quantitative Structure−Property Relationship Models for Early ADME Evaluation in Drug Discovery. 1. Aqueous Solubility. J. Chem. Inf. Comput. Sci..

[90] Klamt A, Eckert F, Hornig M, Beck ME, Bürger T (2002). Prediction of aqueous solubility of drugs and pesticides with COSMO-RS. J. Comput. Chem..

[91] Engkvist O, Wrede P (2002). High-Throughput, In Silico Prediction of Aqueous Solubility Based on One- and Two-Dimensional Descriptors. J. Chem. Inf. Comput. Sci..

[92] Chen X, Cho SJ, Li Y, Venkatesh S (2002). Prediction of aqueous solubility of organic compounds using a quantitative structure–property relationship. J. Pharm. Sci..

[93] Wegner JK, Zell A (2003). Prediction of Aqueous Solubility and Partition Coefficient Optimized by a Genetic Algorithm Based Descriptor Selection Method. J. Chem. Inf. Comput. Sci..

[94] Cheng A, Merz KM (2003). Prediction of Aqueous Solubility of a Diverse Set of Compounds Using Quantitative Structure−Property Relationships. J. Med. Chem..

[95] Yan A, Gasteiger J (2003). Prediction of Aqueous Solubility of Organic Compounds by Topological Descriptors. QSAR Comb. Sci..

[96] Lind P, Maltseva T (2003). Support vector machines for the estimation of aqueous solubility. J. Chem. Inf. Comput. Sci..

[97] Yan A, Gasteiger J, Krug M, Anzali S (2004). Linear and nonlinear functions on modeling of aqueous solubility of organic compounds by two structure representation methods. J. Comput. Aided Mol. Des..

[98] Hou TJ, Xia K, Zhang W (2004). ADME Evaluation in Drug Discovery. 4. Prediction of Aqueous Solubility Based on Atom Contribution Approach. J. Chem. Inf. Comput. Sci..

[99] Fröhlich H, Wegner JK, Zell A (2004). Towards Optimal Descriptor Subset Selection with Support Vector Machines in Classification and Regression. QSAR Comb. Sci..

[100] Votano JR, Parham M, Hall LH, Kier LB, Hall LM (2004). Prediction of aqueous solubility based on large datasets using several QSPR models utilizing topological structure representation. Chem. Biodivers..

[101] Clark M (2005). Generalized Fragment-Substructure Based Property Prediction Method. J. Chem. Inf. Model..

[102] Catana C, Gao H, Orrenius C, Stouten PFW (2005). Linear and nonlinear methods in modeling the aqueous solubility of organic compounds. J. Chem. Inf. Model..

[103] Wassvik CM, Holmén AG, Bergström CAS, Zamora I, Artursson P (2006). Contribution of solid-state properties to the aqueous solubility of drugs. Eur. J. Pharm. Sci..

[104] Schwaighofer A (2007). Accurate Solubility Prediction with Error Bars for Electrolytes:  A Machine Learning Approach. J. Chem. Inf. Model..

[105] Cheung, M., Johnson, S., Hecht, D. & Fogel, G. B. Quantitative structure-property relationships for drug solubility prediction using evolved neural networks. in *2008 IEEE Congress on Evolutionary Computation (IEEE World Congress on Computational Intelligence)* 688–693 (2008). 10.1109/CEC.2008.4630870.

[106] Duchowicz PR, Talevi A, Bruno-Blanch LE, Castro EA (2008). New QSPR study for the prediction of aqueous solubility of drug-like compounds. Bioorg. Med. Chem..

[107] Hughes LD, Palmer DS, Nigsch F, Mitchell JBO (2008). Why Are Some Properties More Difficult To Predict than Others? A Study of QSPR Models of Solubility, Melting Point, and Log P. J. Chem. Inf. Model..

[108] Du-Cuny L, Huwyler J, Wiese M, Kansy M (2008). Computational aqueous solubility prediction for drug-like compounds in congeneric series. Eur. J. Med. Chem..

[109] Obrezanova O, Gola JMR, Champness EJ, Segall MD (2008). Automatic QSAR modeling of ADME properties: blood–brain barrier penetration and aqueous solubility. J. Comput. Aided Mol. Des..

[110] Duchowicz PR, Castro EA (2009). QSPR Studies on Aqueous Solubilities of Drug-Like Compounds. Int. J. Mol. Sci..

[111] Ghafourian T, Bozorgi AHA (2010). Estimation of drug solubility in water, PEG 400 and their binary mixtures using the molecular structures of solutes. Eur. J. Pharm. Sci..

[112] Muratov EN (2010). New QSPR equations for prediction of aqueous solubility for military compounds. Chemosphere.

[113] Jain P, Yalkowsky SH (2010). Prediction of aqueous solubility from SCRATCH. Int. J. Pharm..

[114] Eric S (2010). The importance of the accuracy of the experimental data for the prediction of solubility. J. Serbian Chem. Soc..

[115] Louis B, Agrawal VK, Khadikar PV (2010). Prediction of intrinsic solubility of generic drugs using MLR, ANN and SVM analyses. Eur. J. Med. Chem..

[116] Fatemi M, Heidari A, Ghorbanzadeh M (2010). Prediction of Aqueous Solubility of Drug-Like Compounds by Using an Artificial Neural Network and Least-Squares Support Vector Machine. Bull. Chem. Soc. Jpn..

[117] Salahinejad M, Le TC, Winkler DA (2013). Aqueous solubility prediction: do crystal lattice interactions help?. Mol. Pharm..

[118] McDonagh JL, Nath N, De Ferrari L, van Mourik T, Mitchell JBO (2014). Uniting Cheminformatics and Chemical Theory To Predict the Intrinsic Aqueous Solubility of Crystalline Druglike Molecules. J. Chem. Inf. Model..

[119] Kim S, Jinich A, Aspuru-Guzik A (2017). MultiDK: A Multiple Descriptor Multiple Kernel Approach for Molecular Discovery and Its Application to Organic Flow Battery Electrolytes. J. Chem. Inf. Model..

[120] Coley CW, Barzilay R, Green WH, Jaakkola TS, Jensen KF (2017). Convolutional Embedding of Attributed Molecular Graphs for Physical Property Prediction. J. Chem. Inf. Model..

[121] Cho H, Choi IS (2019). Enhanced Deep-Learning Prediction of Molecular Properties via Augmentation of Bond Topology. ChemMedChem.

[122] Cho, H. & Choi, I. S. Enhanced Deep-Learning Prediction of Molecular Properties via Augmentation of Bond Topology. *Chem Med Chem* **14**, 1604 (2019). 10.1002/cmdc.201900458 31389167

[123] Deng T, Jia G (2020). Prediction of aqueous solubility of compounds based on neural network. Mol. Phys..

[124] Gao P, Zhang J, Sun Y, Yu J (2020). Accurate predictions of aqueous solubility of drug molecules via the multilevel graph convolutional network (MGCN) and SchNet architectures. Phys. Chem. Chem. Phys..

[125] Falcón-Cano G, Molina C, Cabrera-Pérez MA (2020). ADME prediction with KNIME: In silico aqueous solubility consensus model based on supervised recursive random forest approaches. ADMET DMPK.

[126] Shen WX (2021). Out-of-the-box deep learning prediction of pharmaceutical properties by broadly learned knowledge-based molecular representations. Nat Mach Intell.

[127] Tosca EM, Bartolucci R, Magni P (2021). Application of Artificial Neural Networks to Predict the Intrinsic Solubility of Drug-Like Molecules. Pharmaceutics.

[128] Wieder O (2021). Improved Lipophilicity and Aqueous Solubility Prediction with Composite Graph Neural Networks. Molecules.

[129] Chen J-H, Tseng YJ (2021). Different molecular enumeration influences in deep learning: an example using aqueous solubility. Briefings Bioinf.

[130] Panapitiya G (2022). Predicting Aqueous Solubility of Organic Molecules Using Deep Learning Models with Varied Molecular Representations. ACS Omega.

[131] Hou Y, Wang S, Bai B, Chan HCS, Yuan S (2022). Accurate Physical Property Predictions via Deep Learning. Molecules.

[132] Raevsky, O. A., Grigor’ev, V. Y., Polianczyk, D. E., Raevskaja, O. E. & Dearden, J. C. Calculation of aqueous solubility of crystalline un-ionized organic chemicals and drugs based on structural similarity and physicochemical descriptors. *J Chem Inf Model*. **54**, 683–91, 10.1021/ci400692n (2014). 10.1021/ci400692n 24456022

[133] Schaper, K.-J., Kunz, B. & Raevsky, O. Analysis of water solubility data on the basis of HYBOT descriptors. Part 2. *QSAR Comb. Sci*. **22**, 943–958, 10.1002/qsar.200330840 (2003).

